# Assessment of metabolome diversity in black and white pepper in response to autoclaving using MS- and NMR-based metabolomics and in relation to its remote and direct antimicrobial effects against food-borne pathogens†

**DOI:** 10.1039/d4ra00100a

**Published:** 2024-04-03

**Authors:** Mostafa H. Baky, Islam M. Kamal, Ludger A. Wessjohann, Mohamed A. Farag

**Affiliations:** a Department of Pharmacognosy, Faculty of Pharmacy, Egyptian Russian University Badr city 11829 Cairo Egypt; b Microbiology and Immunology Department, Faculty of Pharmacy, Cairo University 11562 Cairo Egypt; c Department of Bioorganic Chemistry, Leibniz Institute of Plant Biochemistry (IPB) Weinberg 3 06120 Halle (Saale) Germany; d Pharmacognosy Department, College of Pharmacy, Cairo University 11562 Cairo Egypt mohamed.farag@pharma.cu.edu.eg

## Abstract

*Piper nigrum* L. (black and white peppercorn) is one of the most common culinary spices used worldwide. The current study aims to dissect pepper metabolome using ^1^H-NMR targeting of its major primary and secondary metabolites. Eighteen metabolites were identified with piperine detected in black and white pepper at 20.2 and 23.9 μg mg^−1^, respectively. Aroma profiling using HS-SPME coupled to GC-MS analysis and in the context of autoclave treatment led to the detection of a total of 52 volatiles with an abundance of β-caryophyllene at 82% and 59% in black and white pepper, respectively. Autoclaving of black and white pepper revealed improvement of pepper aroma as manifested by an increase in oxygenated compounds' level. *In vitro* remote antimicrobial activity against food-borne Gram-positive and Gram-negative bacteria revealed the highest activity against *P. aeruginosa* (VP-MIC 16.4 and 12.9 mg mL^−1^) and a direct effect against *Enterobacter cloacae* at *ca.* 11.6 mg mL^−1^ for both white and black pepper.

## Abstract 2

*Piper nigrum* L. (black and white peppercorn) is one of the most common culinary spices used worldwide.

## Graphical abstract

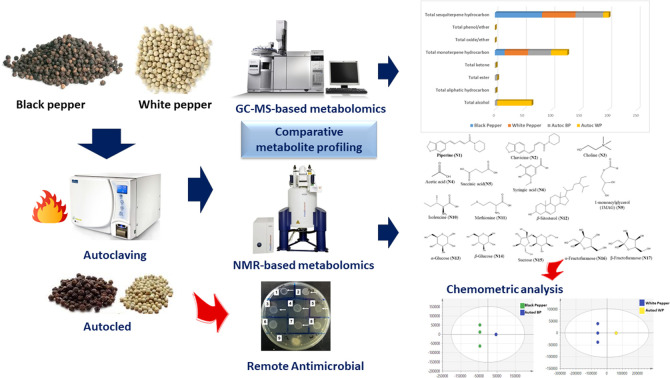

## 1. Introduction

Culinary herbs and spices are valuable food material owing to not only their special aroma but a myriad of nutritional and health benefits.^1^ Black pepper (*Piper nigrum* L.), belonging to the family Piperaceae, is considered as one of the most important spices used worldwide.^2^*P. nigrum* includes different types according to maturity stage such as black pepper, green pepper, and red pepper.^3^ White pepper is produced from *P. nigrum* processing by peeling off its outer black layer.^4^

*P. nigrum* seeds contain a myriad of phytochemicals such as alkaloids, essential oil, lignans, and terpenes, responsible for their biological and nutritional value.^5^*P. nigrum* is used traditionally as a rubefacient, stimulant, appetite stimulant, anti-inflammatory agent, and for treatment of gastric complaints.^6^ Piperine, the major alkaloid in black pepper (5–9%) accounts for its pungency, and further stomachic action.^2,5^ Aside from alkaloids, essential oils are abundant in *P. nigrum* including monoterpenes (*e.g.*, α-pinene, β-pinene, β-myrcene, and limonene), oxygenated monoterpenes (*e.g.*, 1,8-cineole, linalool, terpinen-4-ol, and borneol), sesquiterpenes (*e.g.* β-caryophyllene, humulene, and α-cubebene), and oxygenated sesquiterpenes (*e.g.*, (*E*)-nerolidol, caryophyllene oxide, and bisabolol),^4,7^ that contribute to the seed flavor.

Cooking spices are widely used to impart and improve food flavor and aroma adding to their nutritional value. Likewise, processing of food spices by roasting and heat treatment can improve the quality characteristics of food products including aroma, flavor concurrent with changing their chemical profile.^1^ Autoclaving is increasingly used for food spice sterilization purposes as it can aid to eliminate pathogen microorganisms and affects physicochemical properties, mainly color, flavor, and texture.^8^ Heat processing was found to affect phenolic content and antioxidant capacity of culinary products including black pepper, fennel, cinnamon, cardamom, and clove.^9^ In black pepper, heat processing showed significant loss in piperine level by 16–34%, and likewise observed in case of curcumin in turmeric by 27–53%, and capsaicin in red pepper by 18–36%.^10^ With regards to aroma profile, heat treatment revealed a remarkable change in black pepper aroma by altering its aroma composition with significant increase of monoterpenes: α- and β-pinene, camphene, sabinene, myrcene, α-phellandrene, 3-carene, α-terpinene, *p*-cymene, 1,8-cineole, limonene, γ-terpinene proportions compared to the control.^11^

Owing to their antimicrobial potential, essential oils derived from spices are considered as potential source of food preservatives.^4^ Four pepper oils (*P. longum*, *P. cubeba*, *P. nigrum*, and white pepper) were tested for their antimicrobial potential revealing promising effects against *Helicobacter pylori*, with *P. longum* oil showing the most potential effect with minimum inhibitory concentration (MIC) value at 1.95 μg mL^−1^, comparable to that of clarithromycin antibiotic.^4^ Considering the rich aroma composition in pepper, it can exert remote antibacterial action similar to other herbs and spices,^12^ though not yet revealed in pepper and likely to contribute for its food preservation action. Other biological activities reported in black pepper include antioxidant, anti-inflammatory, cytotoxic, hepatoprotective, digestive, anti-platelet aggregation, and anti-depressant.^13^

Owing to the growth in market demand for spices, a holistic approach is warranted to unveil their quality characteristics and composition.^14^ Recently, metabolomics tools are increasingly employed for profiling and fingerprinting of spices using both targeted and untargeted approaches for quality control purposes. Gas chromatography coupled with mass spectrometry technique (GC-MS) is well adopted for aroma profiling in food products^15^ asides from its high sensitivity. Compared with MS-based, NMR-based metabolomics offers a rapid, robust, and non-destructive tool adopted for the identification and quantification of food chemicals^16^ though less sensitive compared with MS spectroscopy. NMR-spectroscopy offers several measurements including 1D ^1^H NMR and 2D HSQC, TOCSY, and HMBC for identification and quantification of major chemicals^17^ that aid to dissect overlapping NMR peaks observed in 1D-NMR and confirm metabolites identification.

The current study presents the first holistic comparative metabolomics approach in black and white pepper using MS- and NMR-based technologies and visualized using chemometric tools. Whilst NMR provided the targeted quantification of major primary and secondary metabolites in both black and white pepper for standardization purposes, SPME-GC-MS provided an overview of aroma composition and in context to processing to thermal treatment as exemplified by autoclaving for the first time in literature. Further, screening of the remote and direct antimicrobial effects of black and white pepper against 9 food borne bacteria and fungi was also tested using *in vitro* assay revealing inhibitory activity against both Gram-positive and Gram-negative bacteria involved in food contamination.

## 2. Materials and methods

### 2.1. Plant material and autoclave treatment

Authenticated black and white pepper (*Piper nigrum* L.) entire fruits were kindly provided by Dr Ahmed Mediani, Malaysia from the institute of Systems Biology, Universiti Kebangsaan Malaysia, Selangor, UKM Bangi, Malaysia, during October 2022; the plant name followed that listed in plant list website (http://www.theplantlist.org/). The dried fruits were grinded using liquid nitrogen, mortar and pestle and kept at −20 °C till further analyses. A voucher specimen from the fruits was deposited at the College of Pharmacy Herbarium, Cairo University, Cairo, Egypt. Autoclaving was performed by wrapping white and black pepper in aluminum foil and placing them inside an Autoclave ALP Model CNBA-75-1-HH, Japan set at 105 °C for 5 min. Methanol extract was prepared by cold maceration of pepper samples (10 g each) in 100% MeOH (100 mL) with sonication for three times (1 h for each time) and filtration followed by evaporation under reduced pressure at 45 °C to yield dry residue that was kept at −20 °C until further analyses.

### 2.2. SPME and chemicals

Fibers used in SPME volatile extraction including stable flex coated with divinylbenzene/carboxen/polydimethylsiloxane (DVB/CAR/PDMS, 50/30 μm) or PDMS (polydimethylsiloxane) were purchased from Supelco (Oakville, ON, Canada). Volatile and alkane standards were provided from Sigma Aldrich (St. Louis, MO, USA).

### 2.3. SPME-GC-MS volatiles analysis

Freeze dried finely powdered fruits (100 mg) were placed in SPME screw-cap vials (1.5 mL) spiked with 10 μg (*Z*)-3-hexenyl acetate (absent from pepper samples to serve as an internal standard) with fibers inserted manually above and placed in an oven kept at 50 °C for 30 min. HS-SPME analysis of volatile compounds was performed as reported in ref. 18 with slight modifications. The fiber was subsequently withdrawn into the needle and then injected manually into the injection port of a gas chromatography-mass spectrometer (GC-MS). GC-MS analysis was adopted on an Shimadzu GC as reported in.^19,20^ Volatiles separation was made using a DB-5 column (30 m × 0.25 mm i.d. × 0.25 μm film thickness; Supelco) and coupled to a quadrupole mass spectrometer. The interface and the injector temperatures were both set at 220 °C. Volatile elution was carried out using the following gradient temperature program: oven was set at 40 °C for 3 min, then increased to 180 °C at a rate of 12 °C min^−1^, kept at 180 °C for 5 min, finally increased at a rate of 40 °C min^−1^ to 240 °C and kept at this temperature for 5 min. Helium was utilized as a carrier gas with a total flow rate of 0.9 mL min^−1^. For ensuring complete elution of volatiles, SPME fiber was prepared for the next analysis by placing it in the injection port at 220 °C for 2 min. Three different samples for each accession were analyzed under the same conditions to assess biological replicates, and blank runs were made during sample analyses. The mass spectrometer was adjusted to EI mode at 70 eV with a scan range set at *m*/*z* 40–500.

### 2.4. Volatiles identification and multivariate data analyses

Identification of volatile components was performed by comparing their retention indices (RI) in relation to n-alkanes (C6–C20), mass matching to NIST 11.0, WILEY library database and with standards whenever available. Peaks were first deconvoluted using AMDIS software (http://www.amdis.net)^21^ prior to mass spectral matching. Peak abundance data were exported for multivariate data analysis by extraction using MS-Dial software under same conditions cited in.^20,22^ GC-MS files were converted to .netcdf file format using through MS Convert option in Shimadzu program, then to abf files utilizing ABF converter (https://www.reifycs.com/AbfConverter/). In that regard, data analysis was performed using MS dial software (http://prime.psc.riken.jp/compms/msdial/main.html) according to the following parameters: mass range (0–220 Da), MS1 tolerance for alignment (0.015 Da), retention time (0–18 min), minimum peak height (1000), sigma (0.7), accurate mass tolerance (MS) 0.01 Da, and peak area 1000. Peak abundance was exported for multivariate data analysis where final ID and metabolites were Pareto scaled using SIMCA 14.1 (Umetrics, Umea, Sweden) in which the obtained data were subjected to principal component analysis (PCA) and orthogonal partial least squares discriminant analysis (OPLS-DA). PCA was carried out to show the variance of metabolites amongst different samples whilst information on differences in the metabolite composition can be professed by OPLS-DA plot.

### 2.5. Samples preparation for NMR analysis

Samples extraction followed the protocol described in.^19^ All ^1^H-NMR spectra were obtained successively within a 48 h time interval with samples prepared directly before data acquisition. Three biological replicates for each specimen were extracted and analyzed in parallel under the same conditions to assess for biological variance.

### 2.6. NMR data acquisition

All ^1^H-NMR spectra were recorded using an Agilent VNMRS 600 NMR spectrometer as previously reported.^23^ All ^1^H-NMR spectra were recorded on an Agilent VNMRS 600 NMR spectrometer operating at a proton NMR frequency of 599.83 MHz equipped with a 5 mm inverse detection cryoprobe; digital resolution 0.367 Hz/point (32k complex data points); pulse width (pw) = 2.1 μs (30°); relaxation delay = 18 s; acquisition time = 2.0 s; number of transients = 160. Zero filling up to 128k and an exponential window function with lb = 0.4 was used prior to Fourier transformation. 2D-NMR spectra were recorded at a frequency of 599.83 MHz using standard CHEMPACK 6.2 pulse sequences (COSY, HSQC, HMBC) implemented in standard VNMRJ 4.0A spectrometer software. The HSQC experiment was optimized for 1JCH = 146 Hz with DEPT-like editing and 13C-decoupling during acquisition time. The HMBC experiment was optimized for a long-range coupling of 8 Hz; a 2-step 1JCH filter was used (130–165 Hz).

### 2.7. NMR data processing

Spectra were imported to ACD/NMR Manager lab version 10.0 software (Toronto, Canada) and automatically Fourier transformed to ESP files. The spectra were referenced to internal HMDS at 0.062 ppm for ^1^H-NMR and 1.96 ppm for ^13^C-NMR, respectively.^23^ Spectra were imported to ACD/NMR Manager lab version 10.0 software (Toronto, Canada) and automatically Fourier transformed to ESP files. The spectra were referenced to internal HMDS at 0.062 ppm for ^1^H-NMR and 1.96 ppm for ^13^C-NMR, respectively. Spectral intensities were reduced to integrated regions, referred to as buckets, of equal width (0.04 ppm) for all spectral (*δ* 0.4–11.0 ppm) and aromatic (*δ* 5.5–11.0 ppm) regions. The spectral regions corresponding to the residual solvent signals; *δ* 4.90–4.80 (water) and *δ* 3.33–3.28 ppm (methanol), were removed before multivariate analyses. This binning allowed us to evaluate the absolute quantification of the identified metabolites.

### 2.8. Quantification of major metabolites *via*^1^H-NMR

For the quantification of metabolites listed in Table 1 using NMR spectroscopy, the peak area of selected proton signals belonging to the target compounds, and the peak area of the internal standard (HMDS) were integrated manually for all the samples. The equation applied for the calculations was described in ref. 24. The following equation was applied for the calculations:
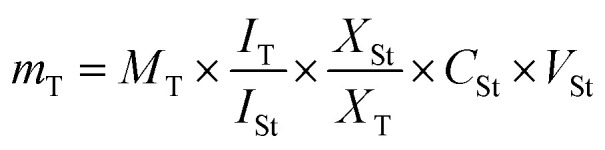
*m*_T_ mass of the target compound in the solution used for ^1^H-NMR measurement (μg), *M*_T_ molecular weight of the target compound (g mol^−1^), *I*_T_ relative integral value of the ^1^H-NMR signal of the target compound, *I*_St_ relative integral value of the ^1^H-NMR signal of the standard compound, *X*_St_ number of protons belonging to the ^1^H-NMR signal of the standard compound. *X*_T_ number of protons belonging to the ^1^H-NMR signal of the target compound, *C*_St_ concentration of the standard compound in the solution used for ^1^H-NMR measurement (mmol l^−1^), *V*_St_ volume of solution used for ^1^H-NMR measurement (ml).

**Table 1:** Resonance assignments with chemical shifts of major constituents identified in 600 MHz ^1^H-NMR, TOCSY, HSQC, and HMBC spectra of pepper methanol extracts in CD_3_OD

Metabolite	Assignment	*δ* ^1^H (ppm)	TOCSY correlations *δ*^1^H (ppm)	HSQC correlations *δ*^13^C (ppm)	HMBC correlations *δ*^13^C (ppm)	Black pepper (μg mg^−1^)	White pepper (μg mg^−1^)
**Alkaloids/nitrogenous**							
Piperine (N1)	C1/3	1.551–1.643 (m)		25.5, 27.8	27.7 (C-2), 44.5 (C-4), 48.1 (C-6)	20.21 ± 4.67	23.94 ± 2.61
	C2	1.679–1.738 (m)		27.7	27.8 (C-3), 44.5 (C-4), 48.1 (C-6)		
	C-4, C-6	3.618–3.636 (m)		44.5, 48.1	25.5 (C-2), 44.5 (C-4), 48.1 (C-6), 167.6 (C-7)		
	C-20	5.961 (s)		102.6	149.7 (C-16–17)		
	C-9	6.611 (d, 14.65 Hz)	7.32	120.6	167.6 (C-7), 144.5 (C-10), 126.3 (C-11)		
	C-11/12	6.803–6.907 (m)		126.3, 140.1	144.5 (C-10), 126.3 (C-11), 106.6 (C-18)		
	C-15	6.783 (d, 8.03 Hz)		109.2	149.7 (C-16), 132.3 (C-13)		
	C-14	6.968 (dd, 1.46, 8.04 Hz)	6.783	123.7	149.7 (C-16), 140.1 (C-12)		
	C-18	7.091 (d, 1.49 Hz)	6.783, 6.9	106.6	149.7 (C-17), 140.1 (C-12), 123.7 (C-14)		
	C-10	7.323 (dd, 14.63, 10.66 Hz)	6.89, 6.63	144.5	167.6 (C-7), 126.3 (C-11), 140.1 (C-12)		
Piperine isomer (chavicine) (N2)	C1/3	1.557–1.641 (m)		25.5, 27.8	27.7 (C-2), 44.5 (C-4), 48.1 (C-6)	7.93 ± 1.04	5.68 ± 1.09
	C2	1.776–1.890 (m)		27.7	27.8 (C-3), 44.5 (C-4), 48.1 (C-6)		
	C-4, C-6	3.607–3.635 (m)		44.5, 48.0	25.5 (C-2), 44.50 (C-4), 48.08 (C-6), 167.66 (C-7)		
	C-20	5.896 s		102.5	149.2 (C-9–10)		
	C-9	6.644 (d, 14.65 Hz)		120.5	167.6 (C-1), 140.1 (C-3), 123.5 (C-5)		
	C-11/12	6.843–6.932 (m)		123.8, 140.1	140.1 (C-3), 120.5 (C-2)		
	C-15	6.893 (d, 10.76 Hz)		106.4	149.3 (C-9), 132.3 (C-6)		
	C-14	6.947 (dd, 1.46, 8.04 Hz)		123.6	149.3 (C-9), 106.6 (C-8)		
	C-18	7.091 (d, 1.49 Hz)		109.3	149.2 (C-10), 132.3 (C-6)		
	C-10	6.803 (dd, 8.15, 9.84 Hz)		144.5	167.6 (C-1), 120.5 (C-2), 140.1 (C-4)		
Choline (N3)	N-(CH_3_)_3_	3.270 (m)*	3.82, 3.64	53.7	53.7 (N-CH_3_), 67.1 (N-CH_2_)		
	N-CH_2_	3.822 (m)*		67.1	53.7 (N-CH_3_)		

**Organic acids/fatty acids**							
Acetic acid (N4)	C-2	1.976 (s)		20.3	172.6 (COO)	0.22 ± 0.06	0.14 ± 0.03
Succinic acid (N5)	C-2 (α,β)	2.529 (s)		35.3	—	0.47 ± 0.06	0.37 ± 0.03
Syringic acid (N6)	C-3/C-5	3.899*		56.3	149.2 (C3/C5)		
	C-2/C-6	7.042*		109.2	123.1 (C1)		
Fatty acids (N7)	C-1	—		179.12			
	C-2	2.228 (m)*		33.89	26.1 (C3), 30.1 (C4), 179.12 (C1)		
	C-3	1.603 (m)*		26.85	30.2 (C3), 125.43, 136.14 (CH=CH)		
	Inner CH_2_	1.285–1.309 brs	0.9, 1.11, 1.55 (terminal CH_3_)	30.1–33.2	23.7, 27.9, 30.7, 32.1 (inner CH_2_)		
	ω-1	1.321*	2.03	28.1	32.9 (inner CH_2_)		
	–CH=CH–	5.338 (t, *J* = 5.59 Hz)	1.32	130.8 (C=C)	28.1 (inner CH_2_)		
			2.03				
	ω-2	0.907 (t, *J* = 7.2 Hz)	1.29	14.4	23.7 (ω-1), 33.03 (ω-2)		
			1.79				
			3.06				
Fatty acid amide (N8)	Inner CH_2_	1.26–1.3	0.9	30.7	23.62, 30.23		
	C-2	2.318	1.61, 1.38	34.8	174.52, 30.23		
1-Monoacylglycerol (1MAG) (N9)	C-3	3.485 (dd, *J* = 5.88, 9.02 Hz)	4.003	64.4, 65.8	25.3, 43.9, 49.8, 69.3		
		3.792 (dd, *J* = 3.31, 5.21 Hz)	3.392	69.3	148.8, 71.7		
	C-2	3.956 (m)	3.751, 3.641	62.7, 83.2	62.7		
	C-1	4.025 (dd, *J* = 5.88, 11.4 Hz)	3.62, 3.76	64.5	63.3		
		4.164 (dd, *J* = 3.58, 12.07 Hz)	4.335	63.1	70.58 (C-2), 164.6 (C=O)		

**Amino acids**							
Isoleucine (N10)	C-5	0.866 (t, *J* = 6.6 Hz)		11.5	22.8 (C-3), 16.6 (C-4)		
	C-6	0.995 (d, *J* = 4.95 Hz)		14.4	22.8 (C-3), 54.5 (C-2)		
	C-4	1.596 m		16.6	22.8 (C-3), 54.5 (C-2)		
	C-3	1.709 m		22.8	14.4 (C-6), 16.6 (C-4)		
	C-2	3.621*		54.5	22.8 (C-3), 167.6 (COO)		
Methionine (N11)	C-6	2.059 (s)		14.4	—	0.67 ± 0.07	0.90 ± 0.08
	C-4	2.697 (t, *J* = 7.5 Hz)		30.6	35.4 (C-3)		
	C-2	2.474*		64.4	173.8 (COO)		
	C-3	1.991*		35.4			

**Sterols**							
β-Sitosterol (N12)	C-18	0.923 (3H, s)		14.4	43.9 (C-13), 55.5 (C-14)	8.14 ± 1.24	3.64 ± 0.34
	C-19	1.195 (3H, s)		20.3	57.2 (C-9), 33.8		
	C26/27	0.850 (d, 6 Hz)		14.4, 20.3	26.9 (C-25)		
	C-3	3.463 (m)		65.8	35.8 (C-2)		
	C-6	5.338 (brt)		125.5	128.9 (C-5), 40.9 (C-4), 30.7 (C-7)		

**Sugars**							
α-Glucose (N13)	C-1	5.095 (d, *J* = 3.6 Hz)	3.35, 3.68	93.9	—	1.48 ± 0.37	1.07 ± 0.81
	C-2	3.35*		73.8	74.7 (C3)		
	C-5	3.68 (d, *J* = 2.4 Hz)		65.8	72.9 (C4)		
β-Glucose (N14)	C-1	4.457 (d, *J* = 7.79 Hz)	3.28, 3.34	98.1	77.9 (C2)	1.07 ± 0.00	1.07 ± 0.64
	C-2	3.288*		77.9	74.8 (C3)		
	C-5	3.344*		73.3	71.6 (C4)		
Sucrose (N15)	C-1	5.019 (d, *J* = 3.85 Hz)	3.42, 3.70	93.6	73.1 (C5), 105.2 (C2′)	2.03 ± 0.59	1.24 ± 0.70
	C-2	3.458 (d, *J* = 1.68 Hz)					
	C-3	3.707*			62.7		
α-Fructofuranose (N16)	C-2	4.711 brs		112.3	35.5	1.36 ± 1.42	6.11 ± 6.49
					49.8		
	C-5	4.028 (d, *J* = 1.2 Hz)		76.78	71.2 (C4)		
β-Fructofuranose (N17)	C-2	4.924 brs		112.3	35.4	1.98 ± 2.22	1.42 ± 1.51
					49.8		
	C-3	4.163 (d, *J* = 5.7 Hz)		76.7	75.6 (C5)		
	C-6	3.734*		63.7	72.9 (C5)		
Glycerol (N18)	C-1	3.575 (m)*		64.6	74.8 (C2)		
	C-2	3.635*		74.8	—		

### 2.9. Statistical analysis

NMR quantification data were analyzed using the Co-Stat version 8 software (Monterey, CA, USA). Data are expressed as mean ± SD of the groups. One-way ANOVA followed by Student–Newman–Keuls tests were used to determine significant differences among pepper sample groups, with 95% confidence limit. Differences were considered statistically significant when *p* ≤ 0.05. Statistical analysis from microbial count test was done using Graphpad prism 6.

### 2.10. Antimicrobial activity

#### 2.10.1. Bacterial strains

Nine standard strains of both bacteria and fungi were tested including: Methicillin-resistant *Staphylococcus aureus* (MRSA USA300), *Enterococcus faecalis* ATCC19433, *Klebsiella pneumoniae* ATCC13883, *Acinetobacter baumannii* AB5075, *Escherichia coli* ATCC87, *Enterobacter cloacae*, and *Salmonella typhi* ATCC35664, *Pseudomonas aeruginosa* PAO1 in addition to *Candida albicans*. The selected bacteria are members of “ESKAPE pathogens” that are commonly known to cause food contamination.

#### 2.10.2. Vapor-phase minimum inhibitory concentration (VP-MIC)

The VP-MIC of ground black and white pepper was performed using airtight box method modified by Sedeek *et al.* (2022).^25^ Briefly, a volume of 15 mL of sterile Mueller–Hinton agar (MHA) was poured in a 10 cm diameter pre-sterilized glass Petri dish. After solidification, MHA surface was inoculated by spotting 10 μL of each of the 9 tested culture suspensions with bacterial counts pre-adjusted to 10^6^ CFU mL^−1^. The spotting of the 9 tested culture suspensions was done side by side in the same Petri dish. The spots were left to dry in a laminar flow cabinet, and then Petri dish was inverted. Each of the tested ground herbs (black pepper or white pepper) were weighed and placed on the cover of the Petri dish. The Petri dish was kept inverted so that inoculated agar was upward and the cover containing the ground pepper was downward. Finally, incubation was done to the inverted Petri dishes at 37 °C for 24 h after sealing with parafilm to prevent evaporation of volatiles. A growth control plate was prepared in the same way by keeping the cover of the Petri dish empty without adding any herbs. For each tested pepper sample, several plates were prepared with different concentrations (40, 20, 9.4, 4.7, 2.4, 1.8, 0.6 mg mL^−1^). The concentration of the tested herbs was calculated as weight/volume by dividing weight of the ground pepper placed in the cover of the Petri dish (mg) by the volume of the airspace in the Petri dish (mL). After incubation, growth was compared in both the tested pepper plates and control plate, and the VP-MIC was determined. The VP-MIC was defined as the least concentration of the tested pepper that resulted in apparent growth inhibition of the tested microorganism when compared to the control. The assay was done at least in three independent replicates, and VP-MIC was reported as mean ± SD (standard deviation). To test the effect of autoclaving on the ground pepper antimicrobial activity, the same experiment was conducted using ground black and white pepper previously autoclaved at 105 °C for 5 min. VP-MIC was calculated as the above-mentioned method.

#### 2.10.3. Minimum inhibitory concentration (MIC) and minimum bactericidal concentration (MBC) for pepper methanol extract

The methanol extracts of both black pepper and white pepper were tested for their MIC and MBC using the broth microdilution method in a 96 well plates for comparison with results from remote effect in Section 2.10.2. For MIC determination, two-fold serially diluted extracts were set in double strength Muller–Hinton broth (40–0.009 mg mL^−1^), dispensed into U-shaped bottom 96-well microplates at a volume of 150 μL. Then, 15 μL of each of the tested bacteria giving positive VP-MIC was added to each well (inoculum size of 10^5^ CFU mL^−1^). The tested bacteria were MRSA *Staphylococcus aureus* USA 300, *Acinetobacter baumannii* AB5075, *Salmonella typhi* ATCC35664, *Enterococcus faecalis* ATCC19433, *Enterobacter cloacae* and *Pseudomonas aeruginosa* PAO1. A control for sterility of the medium was performed with double strength MHB only, while growth control was done with double strength MHB in addition to 15 μL of the inoculum. The microplates were incubated at 37 °C for 24 h. MIC value was determined as the lowest concentration showing no observable bacterial growth.

For MBC determination, microplates from the MIC experiment were used after incubation. A volume of 10 μL was spotted onto Mueller–Hinton agar (MHA) plates from the wells that showed visible inhibition of growth, and incubated for 24 h at 37 °C. The MBC was defined as the minimum concentration of the tested extract without detectable growth on MHA plates of the target organism.^26^ The experiment was done at least in three independent replicates, and MIC was expressed as mean ± SD.

## 3. Results and discussion

The current study aims to assess metabolome variation in black and white pepper in the context of aroma, nutrient, and secondary metabolites profiles, and further in response to autoclaving using MS- and NMR-based metabolomics. Comparison between remote (air based) and direct (extract based) antimicrobial effects against several food borne pathogens was determined to better assess for its food preservative action against bacteria and fungi and in relation to its metabolite profile revealed using NMR and MS techniques.

### 3.1. NMR analysis of black and white pepper fruits' metabolome

**Fig. 1:**
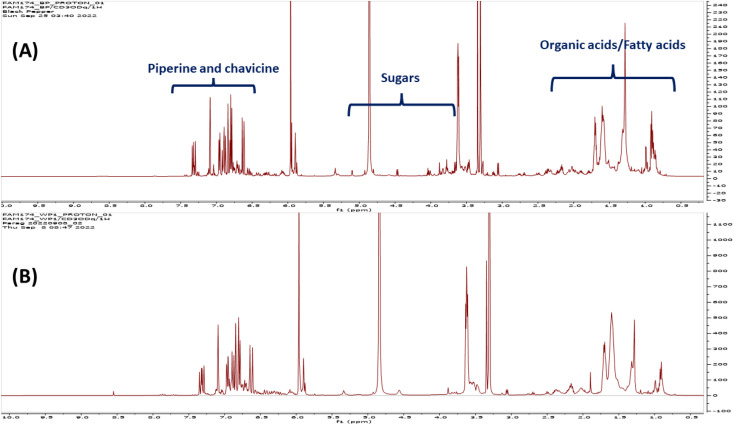
^1^H-NMR spectra of (A) black pepper and (B) white pepper extracts (CD_3_OD) showing compound classes that are observed up-field *vs.* downfield in the NMR spectra.

**Fig. 2:**
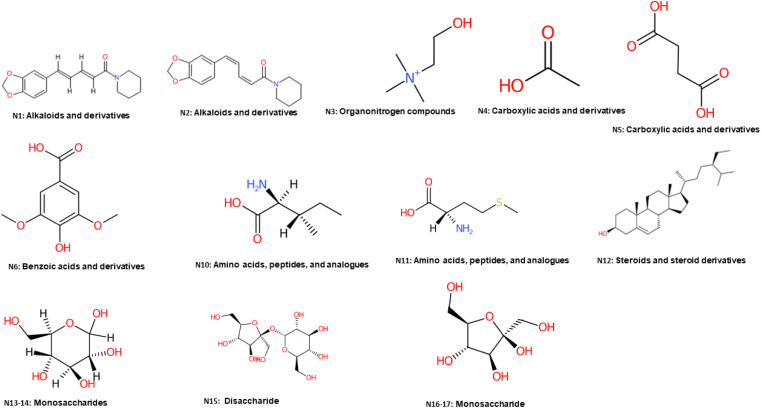
Major identified metabolites in black and white pepper using NMR and classified with ClassyFire.^49^

^1^H-NMR was introduced herein to assess metabolites heterogeneity among black and white pepper, alongside quantification of its major metabolites for future standardization purposes using NMR. ^1^H-NMR signal assignments were further confirmed using 2D-NMR experiments including ^1^H-^1^H TOCSY, ^1^H-^13^C HSQC, and ^1^H-^13^C HMBC to aid in metabolites assignments.^27^ Eighteen metabolites were identified and listed along with their chemical shifts and distribution in black and white pepper, Table 1. ^1^H-NMR spectra from black and white pepper methanol extracts are shown in Fig. 1. ^1^H NMR spectra were divided into two main regions: an upfield region from *δ* 0.5–5.0 ppm for high intensity signals belonging mostly to primary metabolites *viz.* fatty acids (FAs), amino acids, organic acids, and sugars (Fig. 1A). A second downfield region from *δ* 5.0–7.5 ppm was ascribed to secondary metabolites and highly dominated by piperine (N1) and its isomer chavicine (N2) signals (Fig. 1B), structures are listed in Fig. 2.

#### 3.1.1. Alkaloids/nitrogenous compounds

^1^H NMR spectra of black and white pepper showed signals of piperine alkaloids observed between *δ* 1.551 and 7.323 ppm with detailed multiplicity and coupling constants listed in Table 1 & Fig. S1.† All ^1^H NMR signals of piperine alkaloid were in agreement with literature.^2^ 2D-NMR data such as ^1^H-^1^H-TOCSY, ^1^H-^13^C-HSQC, and ^1^H-^13^C-HMBC correlations further confirmed its structure, Table 1. In ^1^H-^1^H-TOCSY, 4 prominent cross peaks were observed at *δ*_H/H_: 6.611/7.323, 6.968/6.783, 7.091/6.783, and 7.323/6.89, Fig. S2.† HSQC spectrum showed 7 cross peaks *δ*_H/C_ indicative of piperine structure as depicted in 5.961/102.6, 6.611/120.6, 6.783/109.2, 6.968/123.7, 7.091/106.6, and 7.323/144.5, Fig. S3,† whereas HMBC spectrum showed cross peaks *δ*_H/C_ with *J*_1–3_ correlations for (N1) alkaloid as evidenced by 7.32/167.6, 126.3, and 140.1, 5.96/149.7 to confirm presence of piperine, Fig. S4 & S5.† In addition to piperine alkaloid, ^1^H NMR spectra showed characteristic signals for its isomer chavicine (N2) from signals appearing at 5.896 (s), 6.644 (d, 14.65 Hz), 6.843–6.932 (m), and 6.803 (dd, 8.15, 9.84 Hz) which was confirmed from cross peaks in 2D NMR spectra. Choline (N3) was identified based on its signals at *δ* 3.27 and 3.88 ppm, assigned based on characteristic N-CH_3_ groups, and confirmed using HSQC and HMBC spectra (Fig. S6†).

#### 3.1.2. Organic acids/fatty acids/sterols

Compared to the abundance of piperine and its isomer in ^1^H NMR, other metabolites were detected at lower levels including FAs, monoacylglycerol, and organic acids (Table 1), and suggestive that pepper present rich source of secondary metabolites as potential functional food or spice. ^1^H NMR spectra showed characteristic signals including olefinic protons (–CH=CH–) of unsaturated fatty acids at *δ* 5.338 ppm (Fig. 1, Table 1). In addition, upfield aliphatic region showed triplet at *δ* 0.907 ppm (*J* = 7.2 Hz) assigned to the terminal methyl group (–CH_3_), whereas methylene groups in fatty chains (–(CH_2_)*n*–) were detected at *δ* 1.285–1.309 ppm (Table 1). Other prominent signals characteristic for FAs were detected such as methylene groups adjacent to the carbonyl moiety (–CH_2_–COOR) appearing at *δ* 2.324 ppm (Table 1). FAs ^1^H NMR data were confirmed using ^1^H-^13^C-HSQC revealing cross peak *δ*_H/C_ between *δ*_H_ 1.26–1.30 with methylene carbon at *δ*_C_ 30.1 indicative of the repeated methylenes in FA chain. Additionally, unsaturation in FA chains was confirmed by the presence of cross peak at *δ*_H/C_ (5.338/130.8) which could be confirmed from HMBC correlation to adjacent methylene at *δ*_H/C_ 2.03/28.1, and the aliphatic methylenes at *δ*_C_ 30.2, Fig. S4.† Moreover, fatty acids were characterized from carbonyl at *δ*_C_ 179.12 and adjacent methylene at *δ*_H/C_ 2.324/34.1. Amide derivatives could be identified from the upfield shift of amide carbon at *δ*_C_ 174.52 and downfield shift of the adjacent methylene proton at *δ*_H_ 2.33 (Table 1, Fig. S4†). ^1^H NMR signals characteristic for glycerol backbone of 1-monoacylglycerol (1-MAG) were assigned in accordance to literature,^2^ in addition to TOCSY, HSQC, and HMBC spectra. 1-MAG ^1^H NMR signals were observed at *δ* 3.485 ppm (dd, *J* = 5.88, 9.02 Hz), *δ* 3.792 ppm (dd, *J* = 3.31, 5.21 Hz), *δ* 3.956 ppm (m), *δ* 4.025 ppm (dd, *J* = 5.88, 11.4 Hz), and *δ* 4.164 ppm (dd, *J* = 3.58, 12.07 Hz) (Table 1).

Characteristic signals for β-sitosterol (N12) were identified at (*δ* 0.923, s; 1.195, s; 0.85, d; 3.463, m; and 5.33, t) and further in 2D spectra HSQC (*δ*_H/C_ 0.923/14.4, 1.195/20.3, and 5.338/125.5), and HMBC (*δ*_H/C_ 5.338/128.9, 40.9, 30.7) in accordance with literature, Fig. S4, S6, and S7.†^28,29^ Other identified primary metabolites included carboxylic acids *i.e.*, acetic (*δ* 1.976 ppm, s), succinic (*δ* 2.529 ppm, s), and syringic acid (*δ* 3.899, 7.042 ppm), with 2D spectra to confirm their assignments.

#### 3.1.3. Amino acids

Isoleucine (N10) as known precursor of piperine alkaloid was identified in^1^H NMR spectrum (*δ* 0.866 ppm, t, *J* = 6.6 Hz; *δ* 0.995 ppm, d, *J* = 4.95 Hz), Table 1. HSQC spectrum showed cross peaks at *δ*_H/C_ 0.866/11.5, 0.995/14.4, and 1.596/16.6, in addition to HMBC at *δ*_H/C_ 0.866/22.8, 16.6 and 0.995/22.8, 54.5. Another major amino acid detected from ^1^H NMR signals at *δ* 2.059 ppm (s) and *δ* 2.697 ppm (t, *J* = 7.5 Hz) was methionine (Table 1). The presence of methionine (N11) was inferred from its characteristic cross peaks in HSQC spectra at *δ*_H/C_ 2.059/14.4, and 2.697/30.6, HMBC cross peaks at *δ*_H/C_ 2.697/35.4 and 3.474/173.8 (COO).

#### 3.1.4. Sugars

The sugar region in ^1^H NMR spectra (*δ* 3.5–5.5 ppm) was rather complex and not rich as expected in most fruits. Major forms included α-glucose (N13), β-glucose (N14), sucrose (N15), α-fructofuranose (N16), and β-fructofuranose (N17) based on respective anomeric protons at *δ* 5.095 (d, *J* = 3.6 Hz), 4.457 (d, *J* = 7.79 Hz), 5.019 (d, *J* = 3.85 Hz), 4.711 (brs), and 4.924 (brs) ppm, (Fig. S8†), and showing HSQC cross-peak correlation at *δ* 93.9, 98.1, 93.6, and 112.3 ppm, respectively (Fig. S8†) in addition to key HMBC cross peaks.^2^

### 3.2. Quantitative NMR of major pepper metabolites

Owing to its universal response to all metabolites, NMR can be used for quantification especially if well resolved discriminatory signals exist^28^*via*^1^H-NMR expressed as μg mg^−1^ dry powder, Table 1. Piperine level in black and white pepper was quantified from its characteristic signal at *δ*_H_ 5.961 (s) detected at 20.2 and 23.9 μg mg^−1^, respectively, and identified as major secondary metabolite.^2^ Unlike piperine, its isomer chavicine was quantified at a much lower level of 7.9, and 5.6 μg mg^−1^ in black and white pepper, respectively. Organic acids were detected at trace levels exemplified by acetic acid at 0.1–0.2 μg mg^−1^, whereas succinic acid was detected at *ca.* 0.4 μg mg^−1^ to likely to contribute to pepper flavor and taste perception. β-Sitosterol^30^ was quantified from its signal at *δ*_H_ 0.923 (s) at much higher level in black pepper (8.1 μg mg^−1^) compared to white pepper (3.6 μg mg^−1^). Regarding sugars, low levels of mono sugars were detected compared to secondary metabolites in both peppers represented by glucose and sucrose at 2.0 and 1.5 μg mg^−1^. In contrast, fructose was quantified at 7.5 and 3.3 μg mg^−1^ in white and black pepper, respectively.

### 3.3. Unsupervised multivariate data analysis of NMR dataset

Both principal component analysis (PCA) score and loading plots were initially constructed to discriminate between black and white pepper metabolite profiles in two attempts, from all spectral width (*δ* 0.4–10.0 ppm), and from the aromatic region only (*δ* 5.5–10.0 ppm) to more focus on secondary metabolites in black and white pepper as illustrated in the heat map (Fig. S9†).

**Fig. 3:**
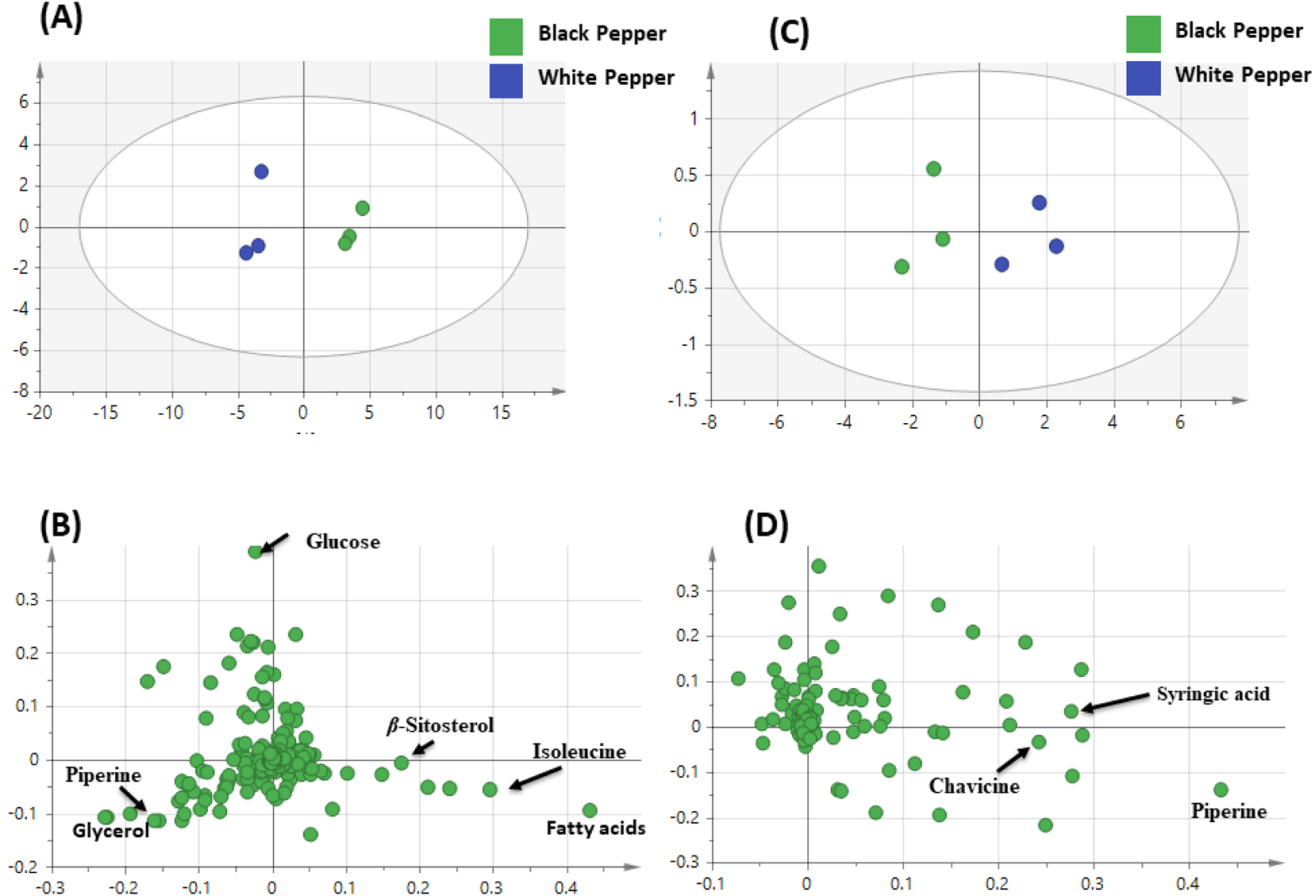
(A) PCA score plot (0.4–10 ppm). (B) Loading plot derived from black pepper modeled against white pepper sample analyzed using ^1^H-NMR (*δ* 0–10.0 ppm), *n* = 3. (C) PCA score plot (5.5–10 ppm). (D) Loading plot derived from black pepper modeled against white pepper sample analyzed by ^1^H-NMR (*δ* 5.5–10.0 ppm), *n* = 3.

The first PCA model based on the full ^1^H-NMR spectrum (*δ* 0–10 ppm) provided an overview of primary and secondary metabolites distribution derived mostly from the aliphatic upfield (*δ* ≤ 5.4 ppm) and the aromatic downfield regions (*δ* > 5.5 ppm), respectively. Based on the full *δ* H scale, PCA score plot accounted for 80% of the total variance (Fig. 3A). An obvious segregation was observed between black pepper at the right side from white pepper at left side alongside PC1. The corresponding loading plot (Fig. 3B) revealed that piperine alkaloid could be recognized as most abundant in white pepper, whereas unsaturated fatty acids, isoleucine, and β-sitosterol were more enriched in black pepper. Likewise, to aid in identifying variation within aromatic region (*δ* H 5.5–10.0 ppm), another PCA model (Fig. 3C) was constructed. The main principal component (PC) to differentiate specimens in PCA *i.e.*, PC1 accounted for 92% of the total variance with black pepper positioned at left side, whereas white pepper segregated towards the right side. PCA Loading plot (Fig. 3D) revealed piperine and its isomer (chavicine) were enriched in white pepper. Such results match the literature which revealed the abundance of piperine in white pepper detected at 2.9% in black *versus* 4.1% in white peppercorn.^31^

### 3.4. Headspace-SPME-GC-MS volatiles analysis of raw and autoclaved pepper

**Fig. 4:**
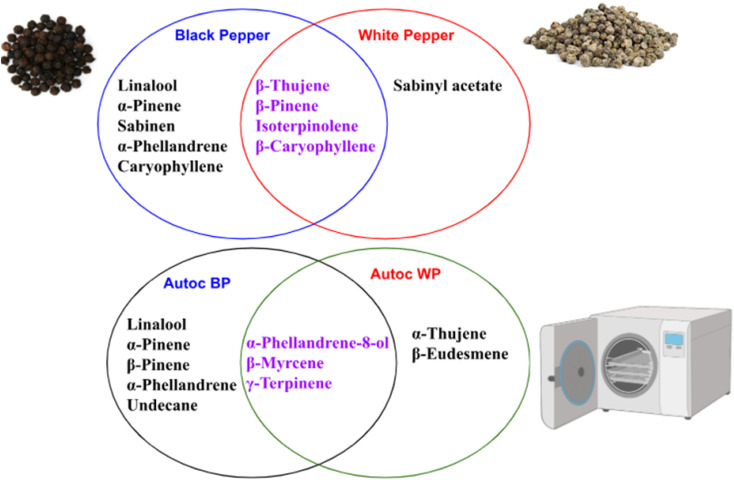
A Venn diagram summarizing the unique and shared metabolites detected in black and white pepper before and after autoclaving.

Assessment of aroma in both white and black pepper pre- and post-autoclaving was performed to account for aroma determinants in pepper as revealed using headspace SPME technique (Fig. 4). A total of 52 volatile peaks were detected in pepper using headspace-solid-phase microextraction (HS-SPME) coupled with GC-MS analysis (Table 2, Fig. S10†). Identified volatiles belonged to several classes *viz.* alcohols, ketones, aliphatic hydrocarbons, oxides/ether, oxygenated monoterpene, esters, phenols/ethers, and monoterpene/sesquiterpene hydrocarbons as discussed in the next subsections.

**Table 2:** Relative percentage of volatiles in raw and autoclaved black and white pepper analyzed *via* GC-MS, *n* = 3

Peak no	Average Rt (min)	Average RI	Name	Class	Black pepper	White pepper	Autoc BP	Autoc WP
1	6.78	890	3-Hexenol	Alcohol	0.01 ± 0.01	—	1.64 ± 1.36	—
20	10.55	1119	4-Thujanol	Alcohol	0.01 ± 0.01	—	0.11 ± 0.13	—
23	10.91	1144	Linalool	Alcohol	0.02 ± 0.02	—	1.47 ± 1.79	—
24	11.03	1152	β-Terpineol	Alcohol	—	—	0.04 ± 0.04	—
28	11.84	1210	α-Phellandrene-8-ol	Alcohol	—	—	0.08 ± 0.13	60.47 ± 16.63
29	12.08	1229	*p*-Cymen-8-ol	Alcohol	—	—	0.20 ± 0.08	—
30	12.20	1237	4-Terpineol	Alcohol	0.02 ± 0.01	—	0.47 ± 0.22	—
**Total alcohol**					**0.06** ± **0.05**	**0.01 ± 0.00**	**4.01 ± 3.77**	**60.47 ± 16.63**
16	10.02	1084	Undecane	Aliphatic hydrocarbon	—	—	1.28 ± 0.51	—
33	13.15	1311	2-Methylundecane	Aliphatic hydrocarbon	—	—	0.13 ± 0.14	0.10 ± 0.04
**Total aliphatic hydrocarbon**					—	—	**1.40 ± 0.66**	**0.10 ± 0.04**
35	—	1340	Sabinyl acetate	Ester	—	0.02 ± 0.02	0.78 ± 0.17	0.18 ± 0.10
42	—	1434	α-Terpinyl acetate	Ester	—	—	3.34 ± 0.31	—
**Total ester**					—	**0.02 ± 0.02**	**4.12 ± 0.48**	**0.18 ± 0.10**
9	9.14	1027	2-Nonanone	Ketone	0.04 ± 0.04	0.05 ± 0.01	1.22 ± 1.24	—
41	14.43	1415	3′-Hydroxyacetophenone	Ketone	—	—	—	0.23 ± 0.19
27	11.59	1192	Camphor	Ketone	—	—	0.02 ± 0.02	0.09 ± 0.05
**Total ketone**					**0.04 ± 0.04**	**0.05 ± 0.01**	**1.24 ± 1.26**	**0.32 ± 0.24**
2	7.36	922	α-Thujene	Monoterpene hydrocarbon	—	—	—	0.21 ± 0.18
3	8.00	959	α-Pinene	Monoterpene hydrocarbon	0.13 ± 0.14	—	4.52 ± 3.33	—
4	8.16	968	β-Thujene	Monoterpene hydrocarbon	0.11 ± 0.10	0.07 ± 0.02	6.37 ± 0.75	—
5	8.72	1002	β-Thujene isomer	Monoterpene hydrocarbon	0.22 ± 0.38	—	4.94 ± 2.34	—
6	8.88	1010	Sabinen	Monoterpene hydrocarbon	0.33 ± 0.17	—	10.80 ± 0.52	—
7	8.99	1018	β-Pinene	Monoterpene hydrocarbon	0.16 ± 0.07	0.15 ± 0.04	1.88 ± 0.11	—
8	9.06	1023	β-Myrcene	Monoterpene hydrocarbon	—	—	0.03 ± 0.03	4.32 ± 1.18
10	9.41	1044	α-Phellandrene	Monoterpene hydrocarbon	0.14 ± 0.24	—	8.39 ± 3.23	—
11	9.53	1052	Isoterpinolene	Monoterpene hydrocarbon	15.57 ± 13.53	40.50 ± 15.81	0.42 ± 0.16	—
12	9.71	1064	Limonene	Monoterpene hydrocarbon	0.05 ± 0.03	0.06 ± 0.02	1.52 ± 1.53	—
13	9.86	1072	*m*-Cymene	Monoterpene hydrocarbon	0.39 ± 0.17	0.23 ± 0.05	0.63 ± 0.67	—
14	9.88	1076	β-Ocimene	Monoterpene hydrocarbon	0.01 ± 0.01	—	0.64 ± 0.15	—
17	10.23	1097	γ-Terpinene	Monoterpene hydrocarbon	—	—	0.01 ± 0.01	20.78 ± 14.06
19	10.47	1111	Terpinolene	Monoterpene hydrocarbon	—	—	0.05 ± 0.06	0.31 ± 0.20
21	10.70	1129	α- Terpinolene	Monoterpene hydrocarbon	0.01 ± 0.01	0.01 ± 0.00	0.26 ± 0.17	—
22	10.76	1135	*p*-Cymenene	Monoterpene hydrocarbon	—	—	0.06 ± 0.07	3.08 ± 2.34
**Total monoterpene hydrocarbon**					**17.12** ± **14.84**	**41.02** ± **15.94**	**40.53** ± **13.12**	**28.69** ± **17.97**
15	9.98	—	Cineole	—	0.01 ± 0.01	—	0.12 ± 0.11	—
**Total oxide/ether**					**0.01** ± **0.01**	—	**0.12** ± **0.11**	—
32	12.69	1274	Methyl mandelate	Phenol/ether	—	—	0.07 ± 0.03	0.17 ± 0.11
**Total phenol/ether**					—	—	**0.07** ± **0.03**	**0.17** ± **0.11**
18	10.36	1105	α-Cubebene	Sesquiterpene hydrocarbon	—	—	0.06 ± 0.06	5.78 ± 1.49
25	11.30	1172	Copaene	Sesquiterpene hydrocarbon	0.04 ± 0.07	—	0.14 ± 0.10	—
26	11.54	1188	β-Elemene	Sesquiterpene hydrocarbon	—	—	—	0.19 ± 0.21
31	12.52	1262	Caryophyllene	Sesquiterpene hydrocarbon	0.30 ± 0.52	—	1.01 ± 0.63	—
34	13.24	1317	α-Humulene	Sesquiterpene hydrocarbon	0.02 ± 0.03	—	0.17 ± 0.18	—
36	13.72	1357	Germacrene D	Sesquiterpene hydrocarbon	0.01 ± 0.01	—	0.06 ± 0.08	0.15 ± 0.07
37	13.93	1371	α-Selinene	Sesquiterpene hydrocarbon	—	—	0.01 ± 0.02	0.24 ± 0.23
38	14.10	1387	β-Farnesene	Sesquiterpene hydrocarbon	0.01 ± 0.02	—	0.30 ± 0.06	3.49 ± 2.04
39	14.21	1397	δ-EIemene	Sesquiterpene hydrocarbon	0.05 ± 0.02	0.06 ± 0.00	0.67 ± 0.40	—
40	14.35	1408	α-Cubebene	Sesquiterpene hydrocarbon	0.01 ± 0.01	—	0.25 ± 0.25	—
43	14.79	1445	Copaene	Sesquiterpene hydrocarbon	0.13 ± 0.08	0.03 ± 0.00	0.33 ± 0.05	—
44	14.91	1454	γ-Gurjunene	Sesquiterpene hydrocarbon	0.02 ± 0.01	0.01 ± 0.00	1.06 ± 1.06	—
45	15.23	1480	α-Gurjunene	Sesquiterpene hydrocarbon	0.01 ± 0.00	—	43.17 ± 5.10	—
46	15.44	1498	β-Caryophyllene	Sesquiterpene hydrocarbon	82.07 ± 13.08	58.76 ± 15.97	0.76 ± 0.52	—
47	15.75	1520	α-Muurolene	Sesquiterpene hydrocarbon	—	—	0.06 ± 0.04	0.06 ± 0.06
48	15.90	1530	α-Bisabolene	Sesquiterpene hydrocarbon	0.06 ± 0.03	0.03 ± 0.00	0.29 ± 0.19	—
49	16.27	1556	d-Germacrene	Sesquiterpene hydrocarbon	0.01 ± 0.01	—	0.09 ± 0.08	0.04 ± 0.04
50	16.40	1564	β-Eudesmene	Sesquiterpene hydrocarbon	—	—	—	0.11 ± 0.15
51	16.47	1570	γ-Selinene	Sesquiterpene hydrocarbon	0.01 ± 0.01	—	0.01 ± 0.01	—
52	16.71	1587	δ-Cadinene	Sesquiterpene hydrocarbon	0.01 ± 0.01	—	0.04 ± 0.04	—
**Total sesquiterpene hydrocarbon**					**82.76** ± **13.90**	**58.90** ± **15.98**	**48.49** ± **8.89**	**10.07** ± **4.29**

#### 3.4.1. Sesquiterpene hydrocarbons

Sesquiterpene amounted for the most abundant class in black and white pepper detected at 82.7 and 58.9%, respectively, with β-caryophyllene (peak 46) as most abundant sesquiterpene at 82.0 and 58.7% in black and white pepper, respectively. β-Caryophyllene was likewise reported at higher level in white pepper oil compared to black pepper at 35.9 and 23.3%, respectively^32^ and in accordance with our SPME results. β-Caryophyllene, an important component of black and white pepper essential oil is recognized for its flavor asides from several health benefits including antioxidant, anti-inflammatory, antimicrobial, hypolipidemic, anticancer, analgesic and antidiabetic activities.^32,33^ On the other hand, a distinct decrease in sesquiterpene hydrocarbons was observed post autoclaving as detected at 48.7% and 10.0% in autoclaved black and white pepper, respectively. Interestingly, β-caryophyllene almost reached trace level in autoclaved black and white pepper owing to its decomposition at elevated temperature.^34^ Compared to relative decrease in β-caryophyllene level, α-gurjunene was detected at much higher level in autoclaved black pepper at 43.1% compared to traces in raw samples. Likewise, α-cubebene and β-farnesene were detected at higher levels in autoclaved white pepper compared to raw fruit at 5.7 and 3.4%, respectively. Hence, autoclaving appeared to alter aroma characteristics of pepper samples based on changes in key aroma compounds.

#### 3.4.2. Monoterpene hydrocarbons

Compared to sesquiterpenes, monoterpene hydrocarbons were detected as the second major class in black and white pepper alongside their autoclaved products at 17.1, 41.0, 41.0, and 29.0%, respectively. Monoterpene hydrocarbons (47–64%) represented the major portion of black pepper oil, while sesquiterpene hydrocarbons (30–47%) predominated in white pepper oil,^35^ and in accordance with our results using SPME. Isoterpinolene (peak 11) was the most abundant at 40.5 and 15.5%, in white and black pepper, respectively likely to originate from thermal isomerization of α-pinene.^36^ Autoclaving of black pepper led to an increase in monoterpenes *viz.* sabinene (peak 6), α-phellandrene (peak 10), β-thujene (peak 4 and 5), α-pinene (peak 3), and limonene (peak 12) detected at 10.8, 8.4, 11.3, 4.5, and 1.5%, respectively. Such increase in monoterpene level in autoclaved black pepper (40.5%) compared to 17.1% in raw fruit is due to thermal degradation of monoterpene hydrocarbons.^37^ Likewise, γ-terpinene (peak 17), β-myrcene (peak 8), and *p*-cymenene (peak 22) were detected at respectively higher levels of 20.7, 4.3, and 3.1% in autoclaved white pepper. Such increase in several monoterpenes in autoclaved pepper infers for thermal changes of monoterpene hydrocarbons.^38^

#### 3.4.3. Alcohols

Unlike sesquiterpene and monoterpenes, alcohols were detected at trace levels in both black and white pepper, while upon autoclaving significant increase was observed in black and white pepper. α-Phellandrene-8-ol (peak 28) constituted the major alcohol in autoclaved white pepper to originate from the thermal isomerization and oxidation of isoterpenoline,^39^ enriched in raw white pepper. α-Phellandrene-8-ol also known as *p*-mentha-1(7),2-dien-8-ol is an oxygenated monoterpene with potential antimicrobial effect^40^ previously reported in pepper essential oil.^4^ Likewise, linalool (peak 23) and 3-hexenol (peak 1) were detected at trace levels in black pepper and slightly increase upon autoclaving to reach 1.5 and 1.6%, respectively. Hence, autoclaving like thermal treatment both appeared to alter aroma profile of culinary products as in pepper, and in accordance with previous reports.^9^

#### 3.4.4. Phenols/oxides/ethers

Oxides/ethers were detected at trace levels in raw and autoclaved black pepper fruits and represented by cineole (peak 15). Cineole level showed likewise increase upon autoclaving indicating that heat treatment can enhance sensory characteristics of black pepper considering its agreeable odor, and to rationalize for pepper heating or roasting treatment prior to its consumption.^10^

#### 3.4.5. Esters/ketones

Esters and ketones were detected at trace levels in both black and white pepper and to increase upon autoclaving to reach 4.1 and 1.2%, respectively. α-Terpinyl acetate with lavender-like pleasant aroma widely used as flavoring agent was the most abundant ester detected in autoclaved black pepper at level 3.3%.^41^ Hence, autoclaving can enhance flavor characteristics of black pepper as in case of roasting. Camphor, a bicyclic ketone with characteristic aroma and antimicrobial properties showed higher level likely due to oxidation of monoterpene hydrocarbon α-pinene with heat treatment.^42^

#### 3.4.6. Aliphatic hydrocarbons

Unlike sesqui- and monoterpene hydrocarbons, aliphatic hydrocarbons were absent in black and white pepper, and to slightly increase upon autoclaving represented by undecane (peak 16) and 2-methylundecane (peak 33).

### 3.5. PCA and OPLS analyses of black and white pepper fruits aroma

#### 3.5.1. PCA analysis of raw and autoclaved black and white pepper fruits' aroma profile

**Fig. 5:**
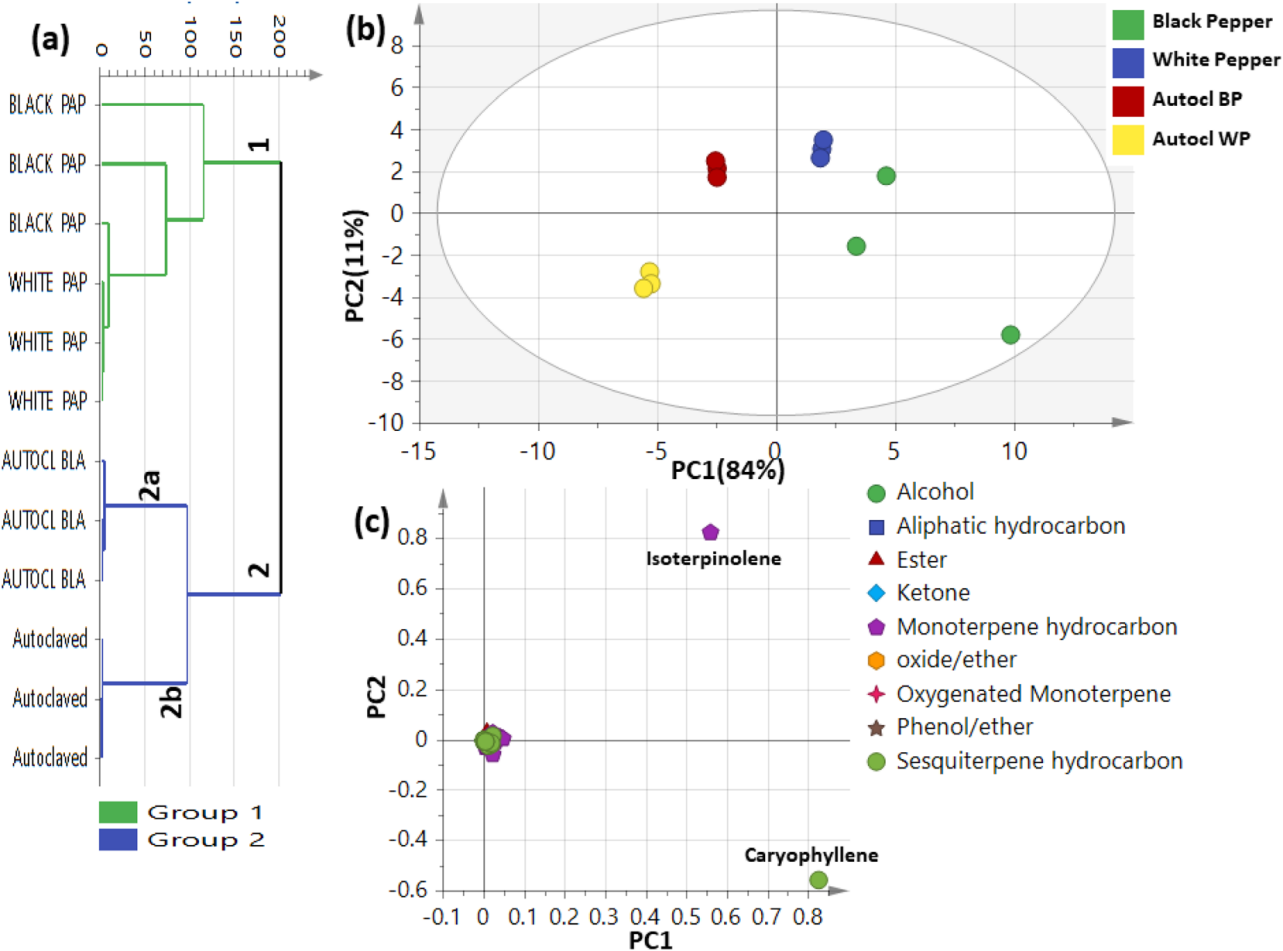
Unsupervised multivariate data analyses of black and white pepper aroma compounds before and after autoclaving detected using GC-MS (*n* = 3). (a) HCA plot. (b) PCA score plot of PC1 *vs.* PC2 scores. (c) The respective loading plot for PC1 and PC2, providing peak assignments. The metabolome clusters are placed in two-dimensional space at the distinct locations defined by two vectors of principal component PC1 = 84% and PC2 = 11%.

Multivariate data analysis using hierarchical cluster analysis (HCA) and principal component analysis (PCA) were used for better assessment of aroma distribution among black and white pepper, and further in context to autoclaving (Fig. 5). HCA depicted a dendrogram in which two distinct clusters (Fig. 5A) were observed, with raw black and white pepper clustered in group 1, whereas autoclaved black and white pepper were positioned into two subdivisions from group 2. The clustering of certain raw and autoclaved pepper together indicated the weakness of HCA model in characterization of volatiles heterogeneity among black and white pepper. A PCA model (Fig. 5B) showed discrimination of black and white pepper clusters at the right side of PC1. In contrast, towards the left side of PC1 showed two clusters: one for autoclaved black pepper at positive side *versus* autoclaved white pepper at the negative side of PC2. The corresponding loading plot Fig. 5C revealed that isoterpinolene (peak 11), a major monoterpene hydrocarbon in raw white pepper, alongside β-caryophyllene (peak 46) were more enriched in raw black pepper and accounting for its segregation.

#### 3.5.2. Supervised OPLS-D analysis of white *vs.* black pepper volatile metabolites

The supervised orthogonal projection to least square discriminant analysis (OPLS-DA) analysis was further employed to classify between black and white pepper. OPLS-DA score plot (Fig. S11A†) revealed segregation of black pepper to the left side while white pepper was segregated toward the right side of score plot. Loading S-plot (Fig. S11B†) revealed that isoterpinolene (peak 11) was enriched in white pepper fruits. OPLS model exhibited total variance coverage of 61% (*R*2 = 0.61) with low prediction power as manifested by *Q*2 = 0.23 and suggestive for no major differences between raw black and white pepper fruits aroma.

#### 3.5.3. Supervised OPLS-DA analysis of raw *versus* autoclaved pepper

A supervised OPLS-DA model (Fig. S12†) was further employed to assess changes in aroma between raw and autoclaved pepper. By constructing a model between raw and autoclaved black pepper (Fig. S12A†) revealing segregation of raw black pepper at the right side *versus* autoclaved black pepper at opposite side with *R*2 = 0.99 and *Q*2 = 0.99 and *p* value of 0.004. Another OPLS-DA model of non-autoclaved white pepper against autoclaved white pepper (Fig. S12B†) showed *R*2 = 0.99 and *Q*2 = 0.98 and *p* value less than 0.05 indicated predictability of the model. The respective loading S-plot (Fig. S12C and D†) revealed that isoterpinolene (peak 11) and β-caryophyllene (peak 46) were both enriched in raw black and white pepper fruits compared with autoclaved ones.

### 3.6. Antimicrobial activity of pepper samples *via* vapor-phase minimum inhibitory concentration (VP-MIC) and MIC of pepper fruit extract

Spices typically present a good source of antimicrobial agents that could be used as food preservative^43,44^ mostly attributed to its essential oil rich composition. Essential oils are though mostly hydrophobic in nature with poor solubility in aqueous solutions and require solvents and emulsifiers for solubilization in aqueous culture media.^45^ To overcome such drawback, VP-MIC was used in this study to assess pepper antimicrobial effect. Another advantage for using VP-MIC lies in its ability to use the solid intact herb to assess its suitability be used as food preservative.^25^ The VP-MIC was examined for the remote inhibition effect of pepper against different resistant bacterial cultures reported to be food borne (Fig. S13A and B†). Interestingly, results showed inhibitory activity of both white and black pepper against both Gram-positive and Gram-negative bacteria using this assay (Table S1†). The highest activity was recorded against *P. aeruginosa* (VP-MIC 16.4 and 12.9 mg mL^−1^) for white pepper and black pepper, respectively. Such results is due to the abundance of sesquiterpene in black and white pepper specially β-caryophyllene which was reported for its flavor asides from antimicrobial properties.^32,33^ However, autoclaving of both black and white pepper resulted in decreased antimicrobial effect as demonstrated by loss of activity against most tested organisms (Table S1†). Such decline in the activity is attributed for the distinct decrease in sesquiterpene hydrocarbons post autoclaving as β-caryophyllene almost reached trace level in autoclaved black and white pepper owing to its decomposition at elevated temperature.^34^ Except for such pattern was sustain of antimicrobial effect post autoclaving against two medically important enteric pathogens (*P. aeruginosa* and *S. typhi*) with though higher VP-MIC values (40 mg mL^−1^) as demonstrated in Table S1.† The decreased antimicrobial activity post autoclaving might be attributed for decrease in key antimicrobial volatiles specially β-caryophyllene which almost reached trace level in autoclaved black and white pepper owing to its decomposition at elevated temperature.^46^ To further compare antimicrobial effect of ground pepper mediated *via* its aroma (remote effect) as analyzed using SPME GCMS, crude extract was prepared and subjected for antimicrobial activity under same conditions as a direct measure of pepper antimicrobial effect better revealed using NMR technique. Pepper extract was further tested for the direct inhibition using microdilution method for the determination of MIC against bacteria especially that showed positive inhibition in VP-MIC (MRSA *Staphylococcus aureus* USA 300, *Acinetobacter baumannii* AB5075, *Salmonella typhi* ATCC35664, *Enterococcus faecalis* ATCC19433, *Enterobacter cloacae* and *Pseudomonas aeruginosa* PAO1). The MIC results using microdilution method of pepper methanol extracts was demonstrated in Table S2.† It should be noted that MIC values in VP-MIC (remote action) were higher than those obtained in the microdilution assay (direct action), which might be due to either the presence of non-volatile antimicrobial compounds in pepper extracts to contribute more to pepper antimicrobial action^47^ or dilution of antimicrobial agent in the headspace above ground pepper powder in remote assay scenario. No bactericidal activity (MBC) was detected for pepper extracts, which suggested that the direct inhibition previously observed in the microplate might be due to a bacteriostatic rather than a bactericidal action.^48^

## 4. Conclusion

The current study presented a comparative NMR and GC-MS based metabolomics approach for *Piper nigrum* analysis including both black and white pepper and in response to autoclaving. NMR fingerprinting of black and white pepper led to the identification of 18 metabolites belonging to alkaloids/nitrogenous, organic/fatty/amino acids, sterols, and sugars among which 11 metabolites were quantified for the first time. Piperine (N1) was the major metabolites quantified in both black and white pepper at comparable levels 20–24 μg mg^−1^, respectively. Furthermore, 52 volatiles belonging to different classes were identified using HS-SPME/GC-MS analysis. Sesquiterpene hydrocarbons amounted as major class in black and white pepper detected at *ca.* 82.7 and 58.9%, respectively represented mostly by β-caryophyllene (peak 46) as most abundant at *ca*. 82 and 58.7%, respectively. A supervised OPLS-DA model of raw and autoclaved pepper fruit revealed distinct segregation between raw and autoclaved pepper indicating the effect of autoclaving on pepper sensory attributes but not efficient to distinguish between raw black and white pepper. Screening for the remote antimicrobial activity of both black and white pepper was tested *in vitro* revealing inhibitory activity against both Gram-positive and Gram-negative bacteria, which suggests that pepper condiment could be used as a natural food preservative. Moreover, the remote antimicrobial effect was retained against the powerful enteric pathogens *P. aeruginosa* and *S. typhi* after autoclaving. However, the remote activity against other bacteria was lost post autoclaving. The direct antimicrobial activity of pepper methanol extract was more powerful than the remote effect, which supports the potential use of pepper in food preservation when it's in direct contact with the food preparation. Future work shall assess other processing methods *i.e.*, pasteurization, or γ-radiation as other methods for sterilization of raw spices. In addition, other spices could be mixed to obtain a wider spectrum of antimicrobial activity to achieve the ecofriendly vision of foods preservation with natural spices as safer options and to benefit from the synergized action of poly herbal formula as typical in most spices. *In vivo* animal models should be the next logical step to evaluate the direct effect of pepper methanol extract in the treatment of enteric pathogens as a possible alternative to conventional antibiotics, and likewise changes to occur on gut microbiota considering the increasing role of their contribution to health status and nutraceuticals effects.

## Author contributions

Mostafa H. Baky: methodology, investigation, formal analysis, writing – review editing. Islam M. Kamal: investigation, writing-original draft, Ludger A. Wessjohann: writing-review & editing Mohamed A. Farag: supervision, conceptualization, investigation, writing – review & editing.

## Conflicts of interest

The authors declare no conflicts of interest.

## Supplementary Material

RA-014-D4RA00100A-s001

## References

[cit1] Serag A., Baky M. H., Döll S., Farag M. A. (2020). RSC Adv..

[cit2] Rivera-Pérez A., Romero-González R., Frenich A. G. (2022). J. Food Compos. Anal..

[cit3] Rivera-Pérez A., López-Ruiz R., Romero-González R., Frenich A. G. (2020). Food Chem..

[cit4] Al-Sayed E., Gad H. A., El-Kersh D. M. (2021). ACS Omega.

[cit5] Ahmad N., Fazal H., Abbasi B. H., Farooq S., Ali M., Khan M. A. (2012). Asian Pac. J. Trop. Biomed..

[cit6] Mgbeahuruike E. E., Yrjönen T., Vuorela H., Holm Y. (2017). S. Afr. J. Bot..

[cit7] da Silva J. K., da Trindade R., Alves N. S., Figueiredo P. L., Maia J. S., Setzer W. (2017). Int. J. Mol. Sci..

[cit8] Zhang H., Tikekar R. V., Ding Q., Gilbert A. R., Wimsatt S. T. (2020). Compr. Rev. Food Sci. Food Saf..

[cit9] Polovka M., Suhaj M. (2010). Food Rev. Int..

[cit10] Suresh D., Manjunatha H., Srinivasan K. (2007). J. Food Compos. Anal..

[cit11] SádeCká J. (2010). Czech J. Food Sci..

[cit12] Taleb M. H., Abdeltawab N. F., Shamma R. N., Abdelgayed S. S., Mohamed S. S., Farag M. A., Ramadan M. A. (2018). Molecules.

[cit13] Verma V. M. , Medicinal Plants: From Farm to Pharmacy, 2019, pp. 111–127

[cit14] Baky M. H., Shamma S. N., Khalifa M. R., Farag M. A. (2022). Metabolites.

[cit15] Baky M. H., Shamma S. N., Xiao J., Farag M. A. (2022). Food Chem..

[cit16] Nagana Gowda G. and Raftery D., Applications, 2021, pp. 19–37 10.1007/978-3-030-51652-9_2 PMC8816450 33791972

[cit17] Mahrous E. A., Farag M. A. (2015). J. Adv. Res..

[cit18] Baky M. H., Elsaid M. B., Farag M. A. (2022). Phytochemisty.

[cit19] Farag M. A., Rasheed D. M., Kamal I. M. (2015). Food Res. Int..

[cit20] Farag M. A., Ali S. E., Hodaya R. H., El-Seedi H. R., Sultani H. N., Laub A., Eissa T. F., Abou-Zaid F. O., Wessjohann L. (2017). Molecules.

[cit21] Afifi S. M., El-Mahis A., Heiss A. G., Farag M. A. (2021). ACS Omega.

[cit22] El-Hawary E. A., Zayed A., Laub A., Modolo L. V., Wessjohann L., Farag M. A. (2022). Antioxidants.

[cit23] Otify A. M., Serag A., Porzel A., Wessjohann L. A., Farag M. A. (2022). Food Anal. Methods.

[cit24] Serag A., Zayed A., Mediani A., Farag M. A. (2023). Sci. Rep..

[cit25] Sedeek M. S., Afifi S. M., Mansour M. K., Hassan M., Mehaya F. M., Naguib I. A., Abourehab M. A., Farag M. A. (2022). Molecules.

[cit26] Serrano I., Alhinho B., Cunha E., Tavares L., Trindade A., Oliveira M. (2023). Life.

[cit27] Farag M. A., Baky M. H., Morgan I., Khalifa M. R., Rennert R., Mohamed O. G., El-Sayed M. M., Porzel A., Wessjohann L. A., Ramadan N. S. (2023). RSC Adv..

[cit28] Farag M. A., Shakour Z. T., Lübken T., Frolov A., Wessjohann L. A., Mahrous E. (2021). J. Pharm. Biomed. Anal..

[cit29] Otify A. M., Serag A., Porzel A., Wessjohann L. A., Farag M. A. (2022). Food Anal. Methods.

[cit30] Yadav P., Chauhan C., Singh S., Banerjee S., Murti K. (2022). Curr. Bioact. Compd..

[cit31] Liu Z.-X., Xiong S.-R., Tang S.-H., Wang Y., Tan J. (2023). Food Res. Int..

[cit32] Myszka K., Schmidt M. T., Majcher M., Juzwa W., Czaczyk K. (2017). LWT--Food Sci. Technol..

[cit33] Hashiesh H. M., Meeran M. N., Sharma C., Sadek B., Kaabi J. A., Ojha S. K. J. N. (2020). Nutrients.

[cit34] Lee H.-Y., Ko M.-J. (2021). Food Sci. Biotechnol..

[cit35] Dosoky N. S., Satyal P., Barata L. M., da Silva J. K. R., Setzer W. (2019). Molecules.

[cit36] Afifi S. M., El-Mahis A., Heiss A. G., Farag M. A. (2021). ACS Omega.

[cit37] Anikeev V. J. F. (2010). Flavour Fragrance J..

[cit38] Sánchez-Velandia J. E., Becerra J.-A., Mejía S. M., Villa A. L., Martínez O F. (2021). ACS Omega.

[cit39] Tibbetts J. D., Bull S. D. (2021). Adv. Sustainable Syst..

[cit40] Dvorakova M., Valterova I., Saman D., Vanek T. J. M. (2011). Molecules.

[cit41] Vaičiulytė V., Ložienė K., Švedienė J., Raudonienė V., Paškevičius A. J. M. (2021). Molecules.

[cit42] Lusi R. F., Perea M. A., Sarpong R. (2022). Acc. Chem. Res..

[cit43] Souza V. V. M. A., Almeida J. M., Barbosa L. N., Silva N. C. C. (2022). J. Essent. Oil Res..

[cit44] Khorrami S., Kamali F., Zarrabi A. (2020). Biocatal. Agric. Biotechnol..

[cit45] Kloucek P., Smid J., Frankova A., Kokoska L., Valterova I., Pavela R. (2012). Food Res. Int..

[cit46] Ge S., Chen Y., Ding S., Zhou H., Jiang L., Yi Y., Deng F., Wang R. (2020). J. Sci. Food Agric..

[cit47] Van Vuuren S., Suliman S., Viljoen A. (2009). Lett. Appl. Microbiol..

[cit48] Khorrami S., Kamali F., Zarrabi A. (2020). Biocatal. Agric. Biotechnol..

[cit49] Djoumbou Feunang Y., Eisner R., Knox C., Chepelev L., Hastings J., Owen G., Fahy E., Steinbeck C., Subramanian S., Bolton E. (2016). J. Cheminf..

