# Supplementary material for: Assessment of metabolome diversity in black and white pepper in response to autoclaving using MS- and NMR-based metabolomics and in relation to its remote and direct antimicrobial effects against food-borne pathogens
Source: RSC Adv. 2024 Apr 3;14(15):10799–813. doi: 10.1039/d4ra00100a (PMC10989240; doi:10.1039/d4ra00100a)
Supplement: RA-014-D4RA00100A-s001 [file RA-014-D4RA00100A-s001.pdf]

Electronic Supplementary Material (ESI) for RSC Advances.
This journal is © The Royal Society of Chemistry 2024

Assessment of metabolome diversity in black and white pepper seeds in response to autoclaving using MS- and NMR-based metabolomics and in relation to its remote and direct antimicrobial effects against food borne pathogens

Mostafa H. Baky[1a], Islam M. Kamal[2a], Ludger A. Wessjohann[3], Mohamed A. Farag [4*]

[1] Department of Pharmacognosy, Faculty of pharmacy, Egyptian Russian University, Badr city, 11829, Cairo, Egypt.

[2]Microbiology and Immunology department, Faculty of Pharmacy, Cairo University,11562 Cairo, Egypt.

[3]Department of Bioorganic Chemistry, Leibniz Institute of Plant Biochemistry (IPB), Weinberg 3, 06120, Halle (Saale), Germany

[4] Pharmacognosy Department, College of Pharmacy, Cairo University, 11562 Cairo, Egypt.

[a]Equal contribution

*Corresponding author at: Cairo University, College of Pharmacy, Department of Pharmacognosy, Egypt.
E-mail addresses: mohamed.farag@pharma.cu.edu.eg, (M.A. Farag).

Table S1: Vapor-phase antimicrobial activity of the tested ground pepper n=3, Average ± SD

	Vapor-phase minimum inhibitory concentration (VP-MIC) mg/mL								
	MRSA USA300	*Acinetobacter baumannii AB5075*	*Salmonella typhi ATCC35664*	*Enterococcus faecalis ATCC19433*	*Enterobacter cloacae*	*Escherichia coli ATCC87*	*Pseudomonas aeruginosa PAO1*	*Klebsiella pneumoniae ATCC13883*	*Candida albicans*
**White pepper**	33.3 ± 11.5	40 ± 0	33.3 ± 11.5	26.6 ± 11.5	20 ± 0	-	16.4 ± 6.1	-	-
**White pepper (autoclaved)**	LA	LA	40 ± 0	LA	LA	-	40 ± 0	-	-
**Black pepper**	26.6 ± 11.5	40 ± 0	23.1 ± 15.5	16.4 ± 6.1	12.9 ± 6.1	-	12.9 ± 6.1	-	-
**Black pepper (autoclaved)**	LA	LA	LA	LA	LA	-	40 ± 0	-	-

(-) indicates that there was no antimicrobial activity detected under the tested conditions

(LA) Indicates the loss of activity after autoclaving.

Table S2: Minimum inhibitory concentration of pepper methanol extracts using microdilution method, n=3, Av±SD.

	Minimum inhibitory concentration (MIC) mg/mL					
	MRSA USA300	*Acinetobacter baumannii* AB5075	*Salmonella typhi* ATCC35664	*Enterococcus faecalis* ATCC19433	*Enterobacter cloacae*	*Pseudomonas aeruginosa* PAO1

**White pepper extract**	6.6 ± 2.8	26.6 ± 11.5	33.3 ± 0	16.6 ± 5.7	11.6 ± 7.6	20 ± 0
**Black pepper extract**	2.9 ± 1.9	20.0	33.3 ± 0	13.3 ± 5.7	11.6 ± 7.6	16.6 ± 5.7

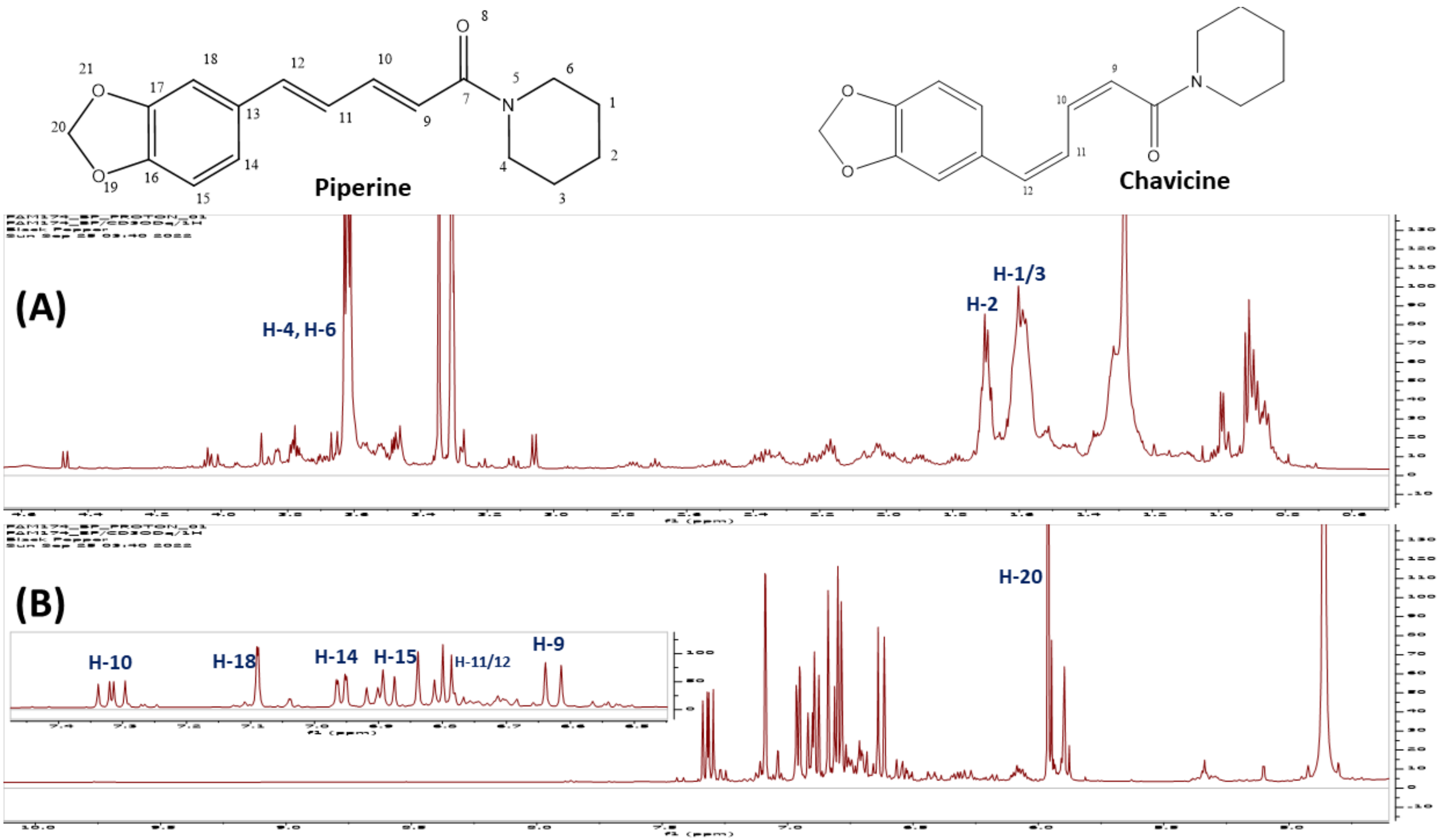

Figure S1. Signal assignment of the ^{1}H-NMR markers for piperine alkaloid

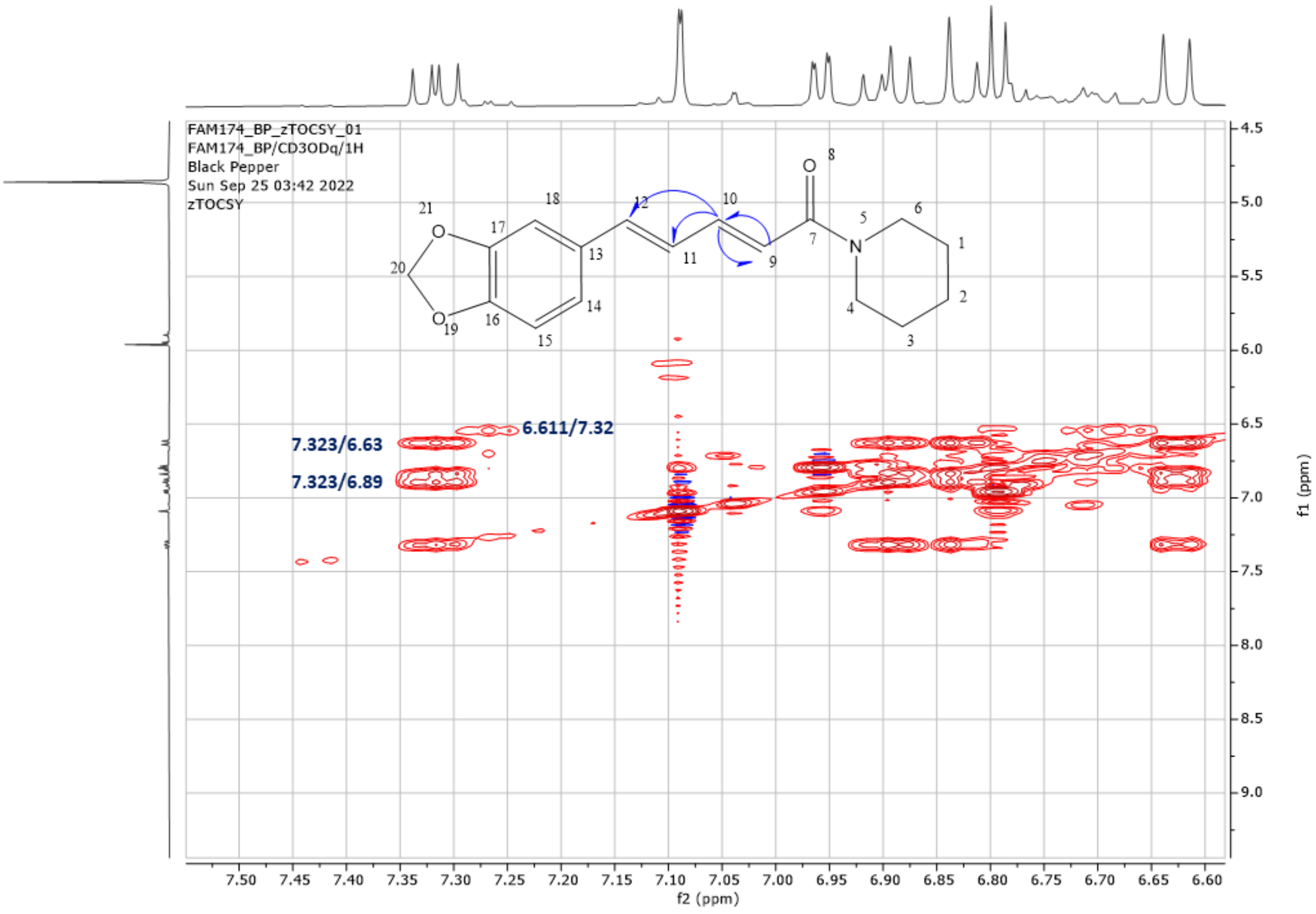

Figure S2. ^{1}H-^{1}H correlations observed in the TOCSY spectrum for piperine

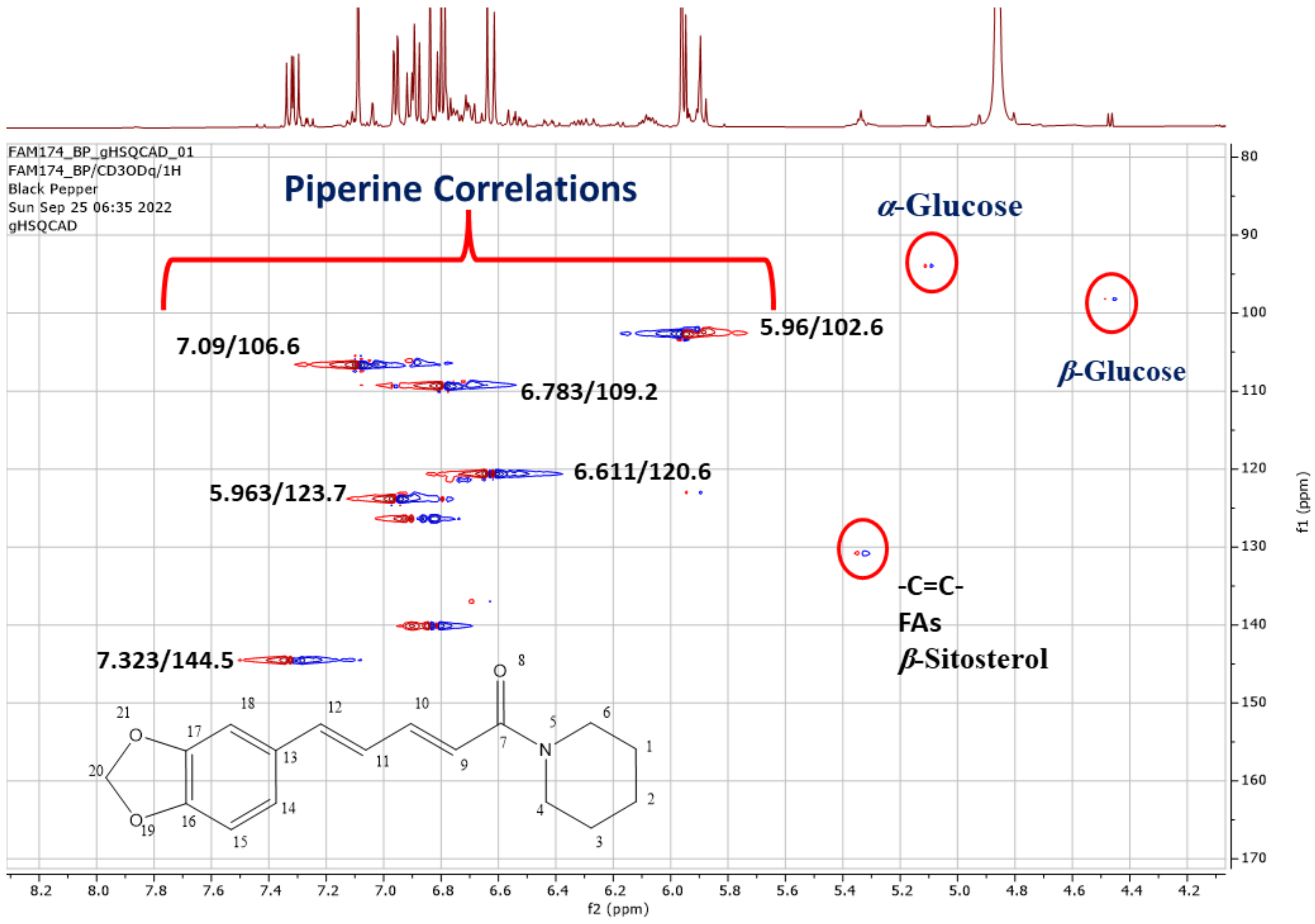

Figure S3. ^{1}H-^{13}C correlations observed in the HSQC spectrum for piperine

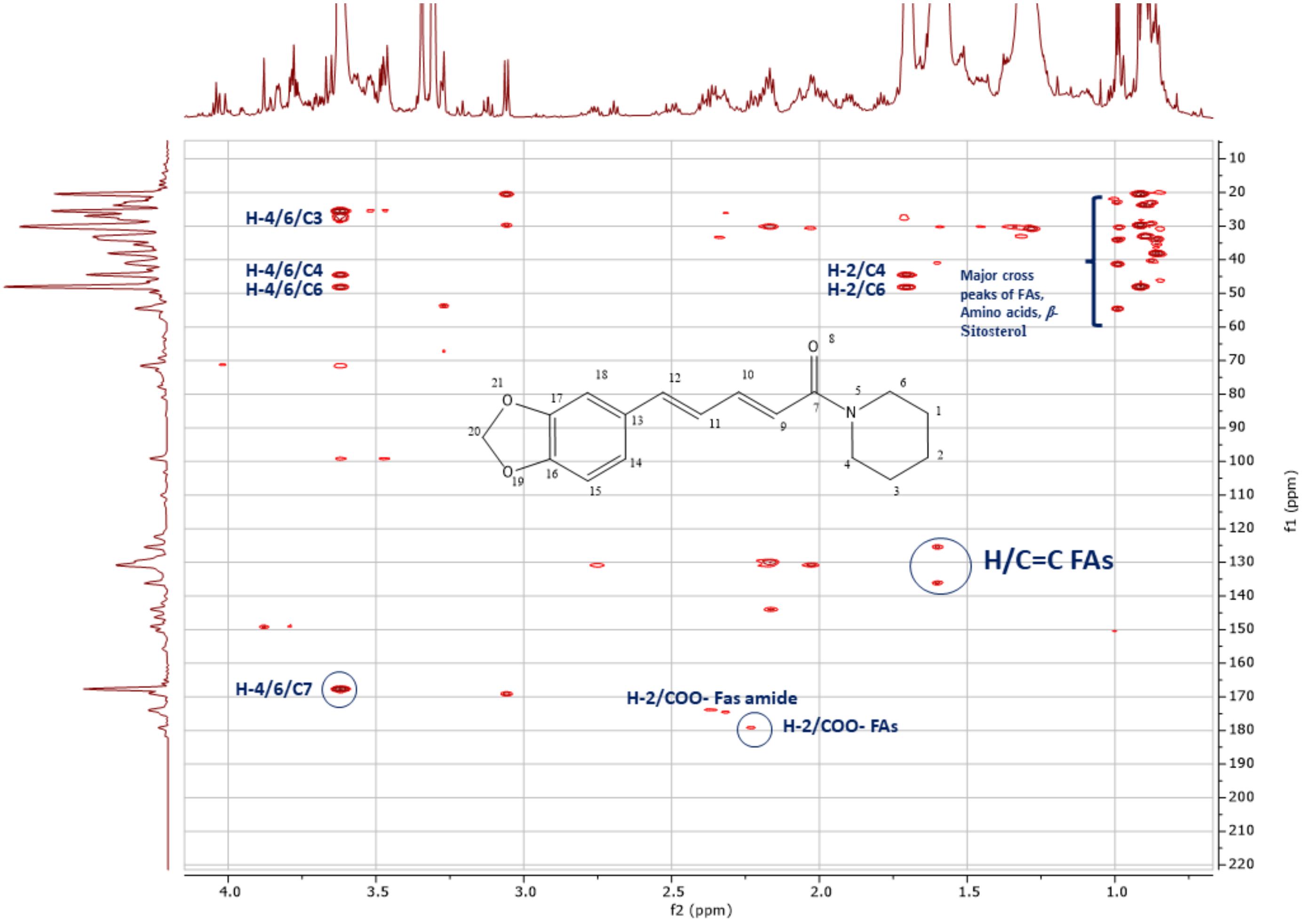

Figure S4. ^{1}H-^{13}C correlations observed in the HMBC spectrum identified metabolites

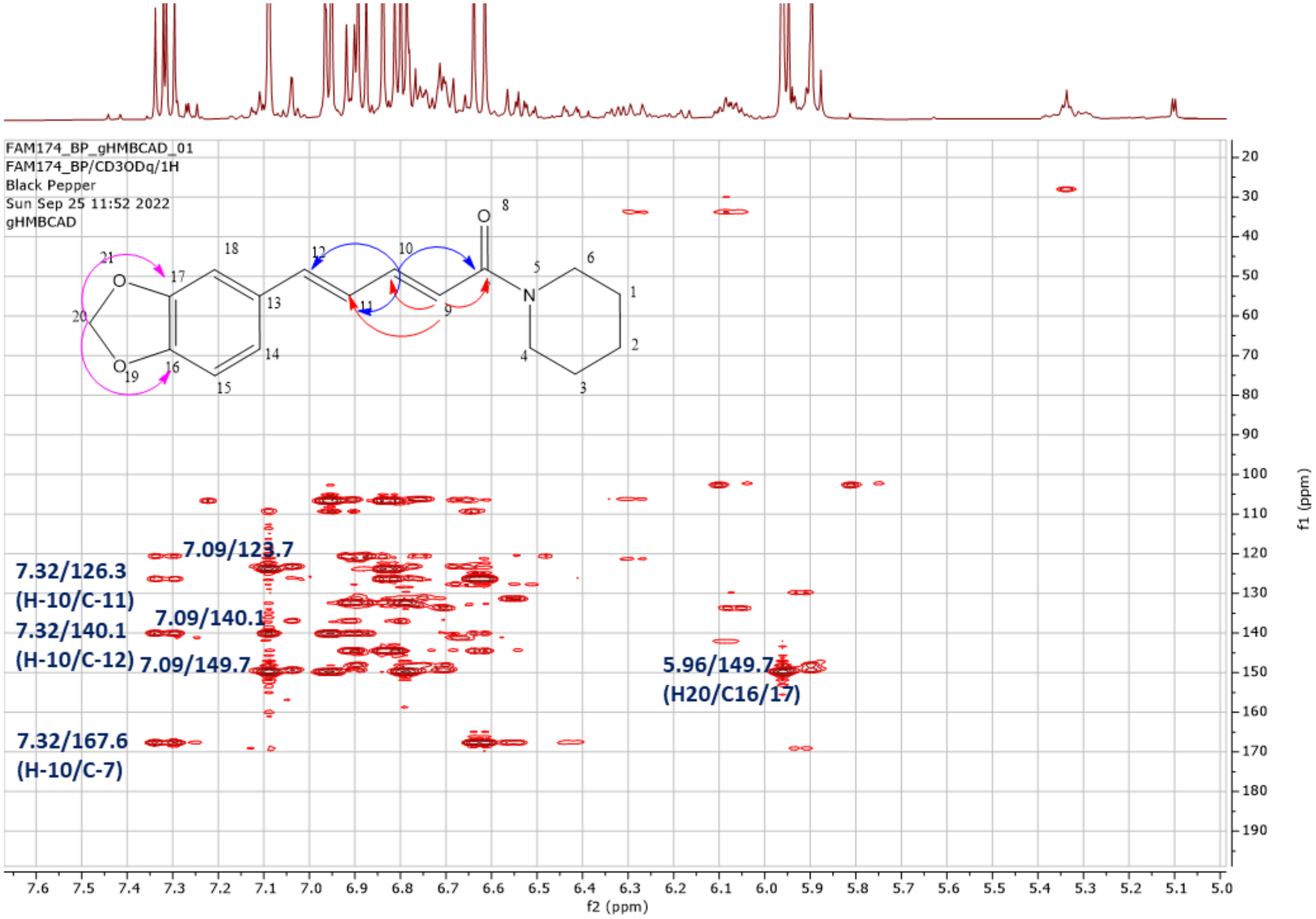

Figure S5. ^{1}H-^{13}C correlations observed in the HMBC spectrum for piperine

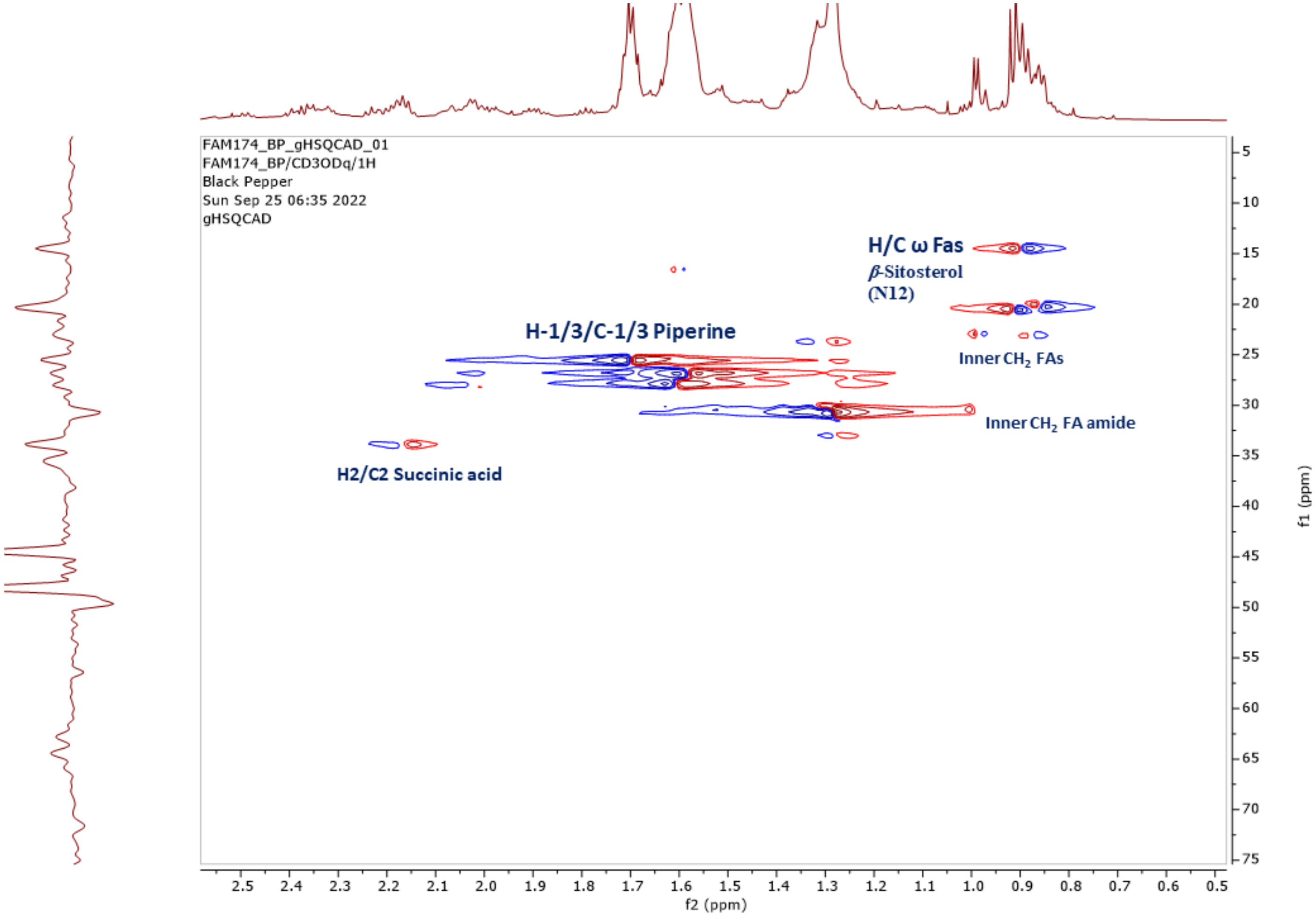

Figure S6. ^{1}H-^{13}C correlations observed in the HSQC spectrum for major primary metabolites

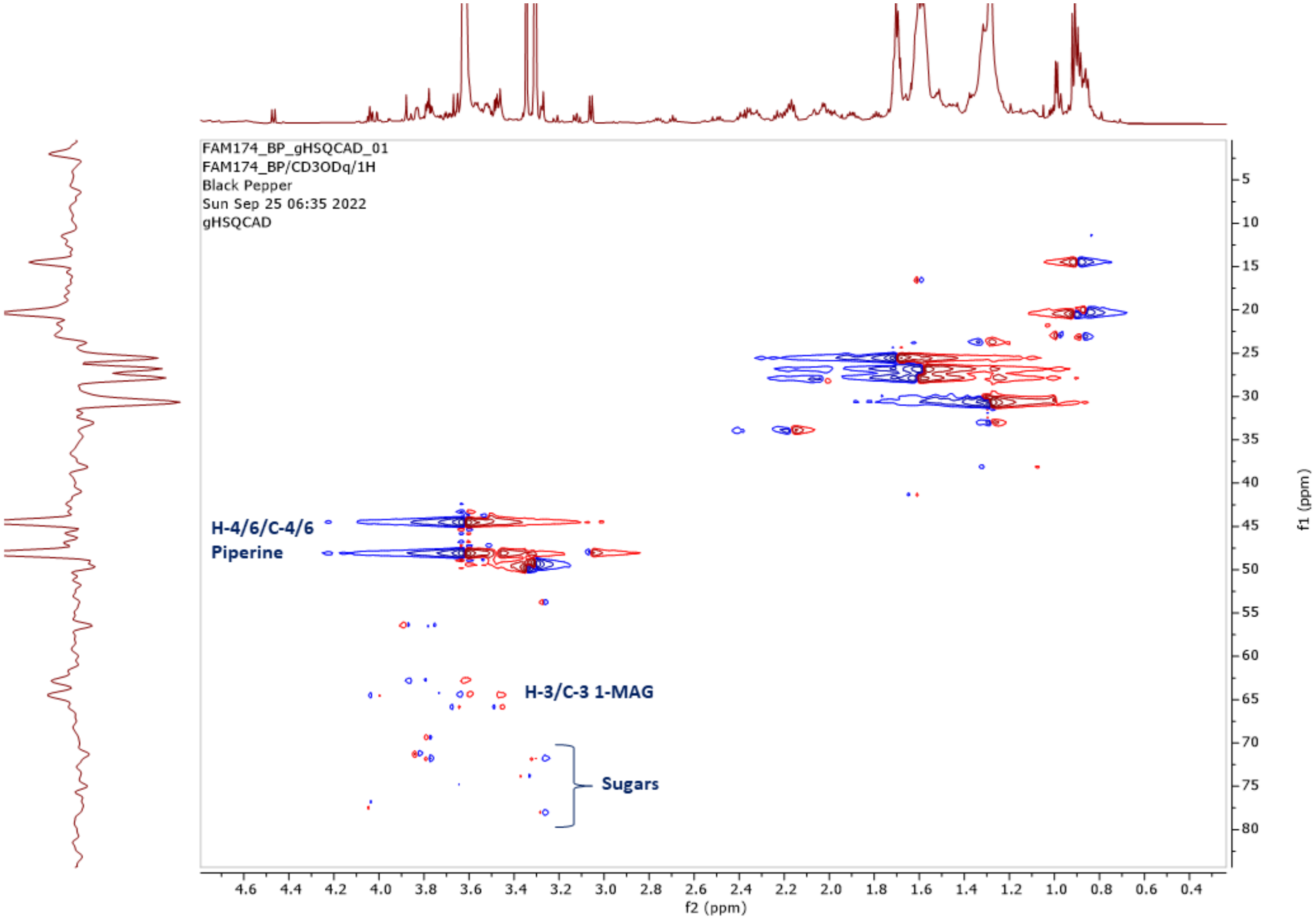

Figure S7. ^{1}H-^{13}C correlations observed in the HSQC spectrum for major primary metabolites

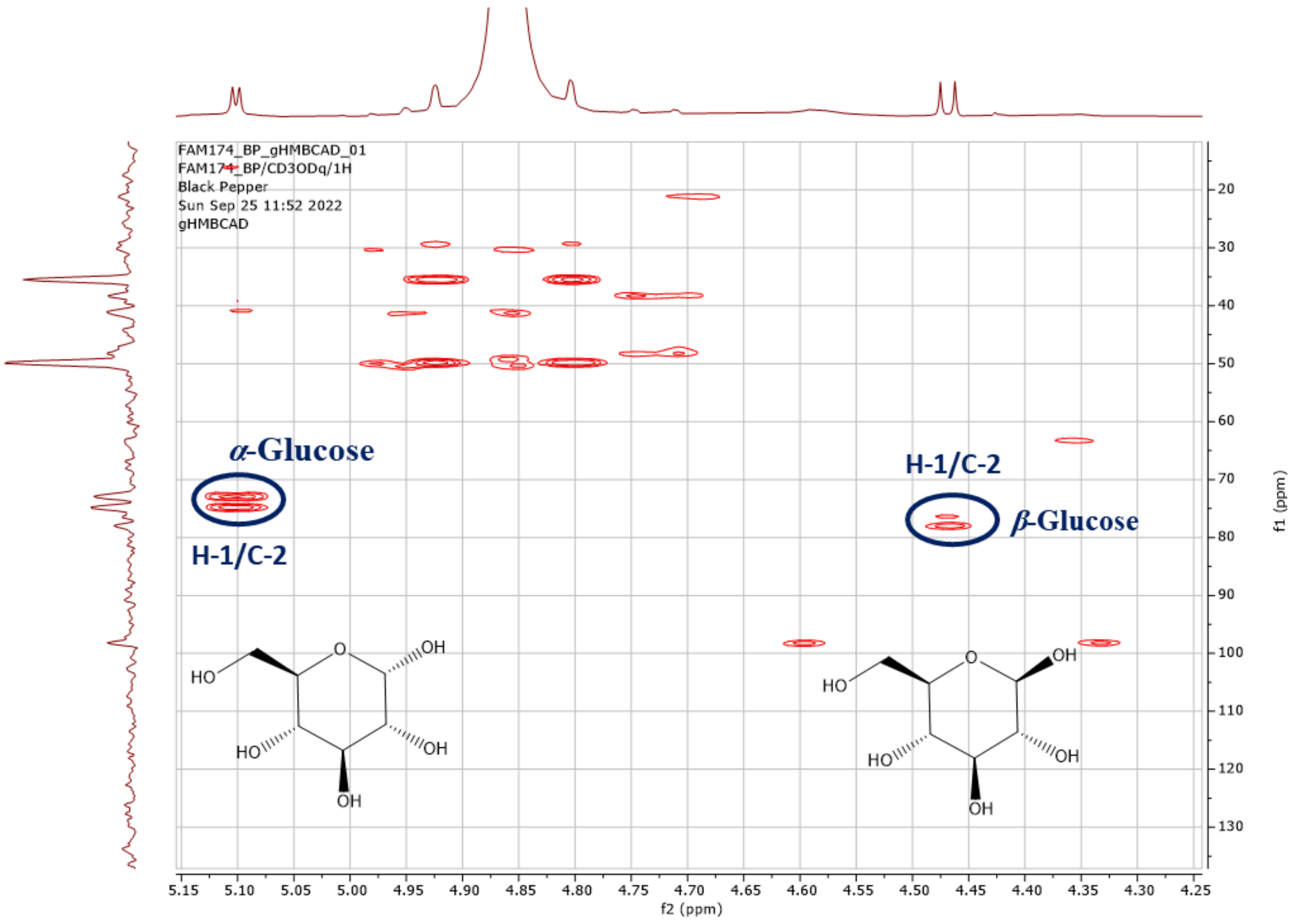

Figure S8. ^{1}H-^{13}C correlations observed in the HMBC spectrum for major primary metabolites

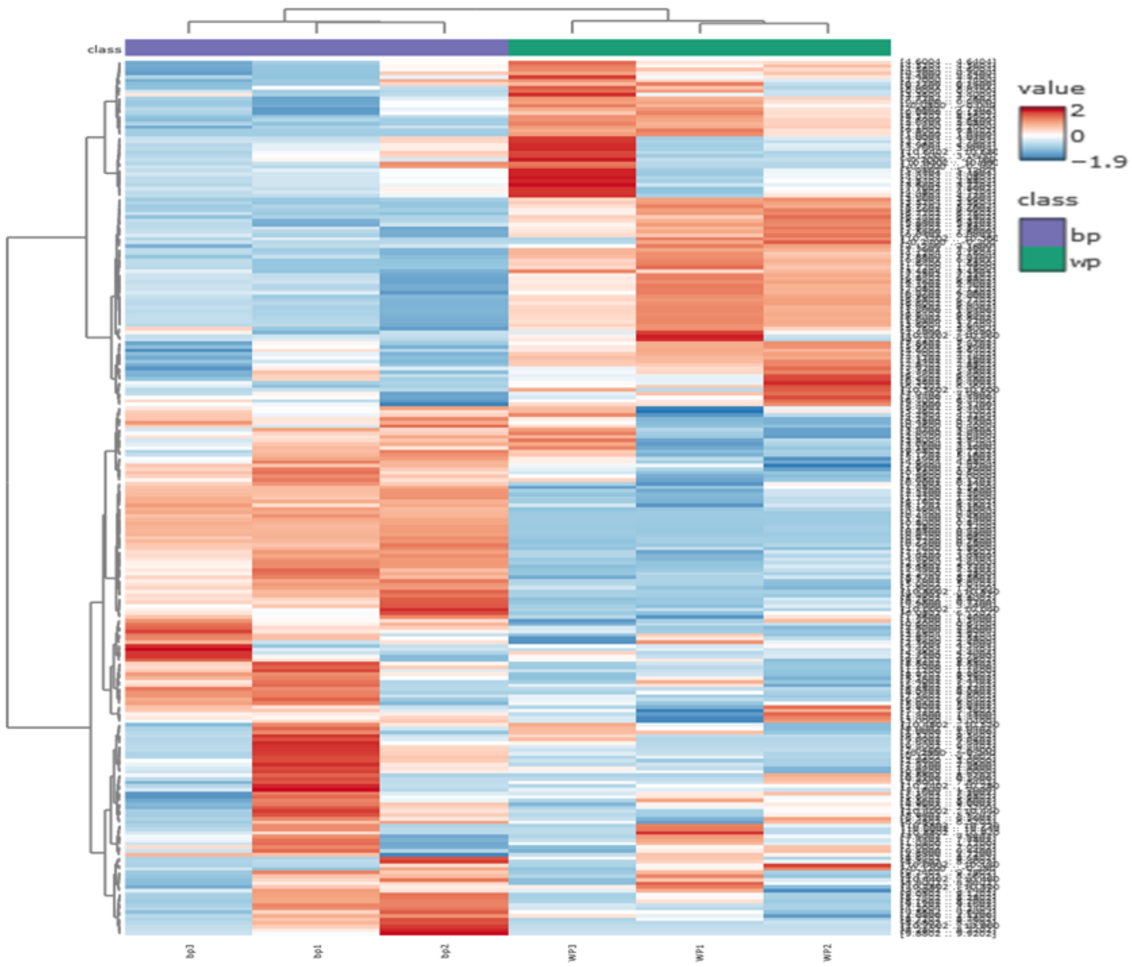

Figure S9. A heat map for identified metabolites in black and white pepper as extracted from NMR Table 1

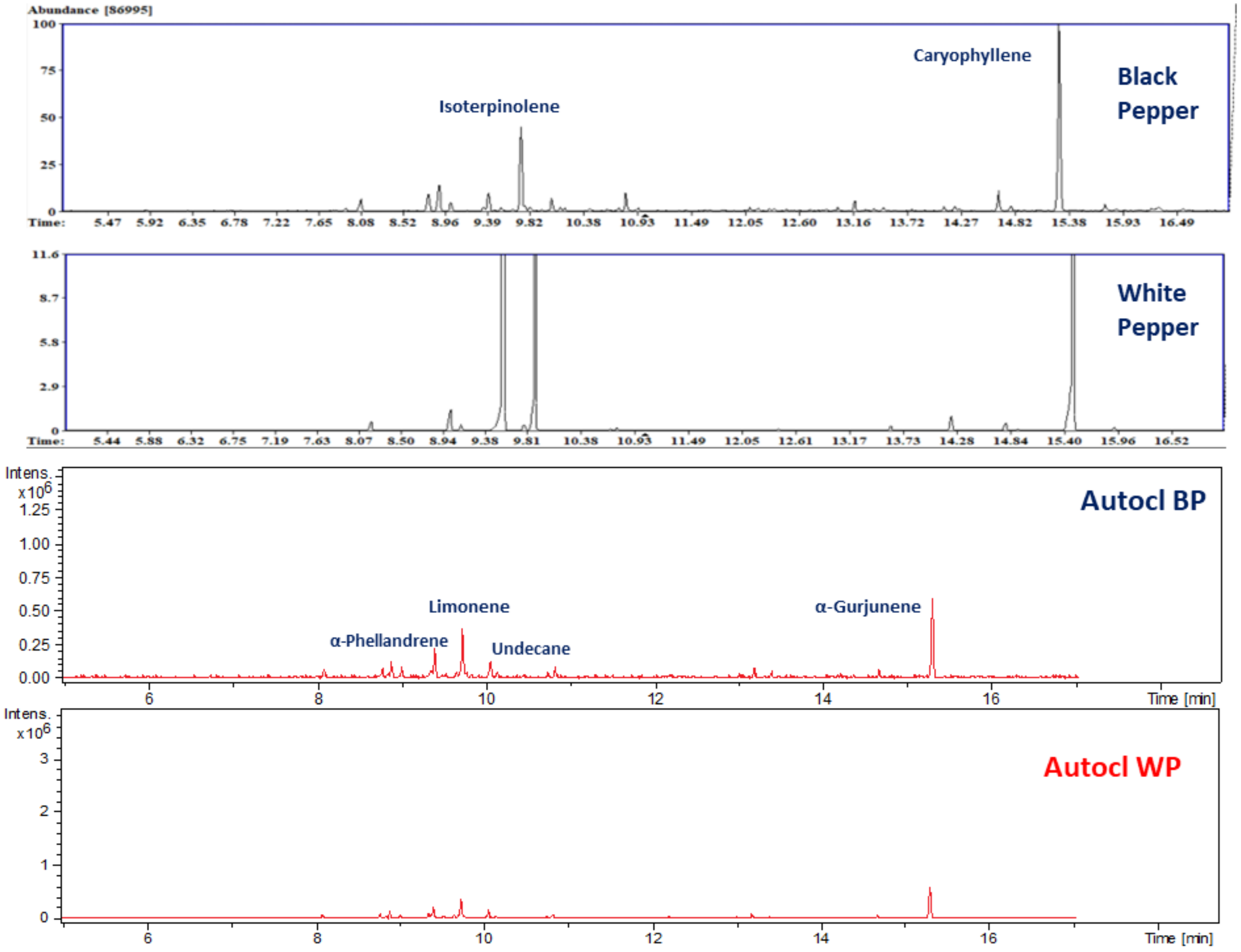

Figure S10. Representative GC–MS chromatograms of volatile constituents of black and white pepper.

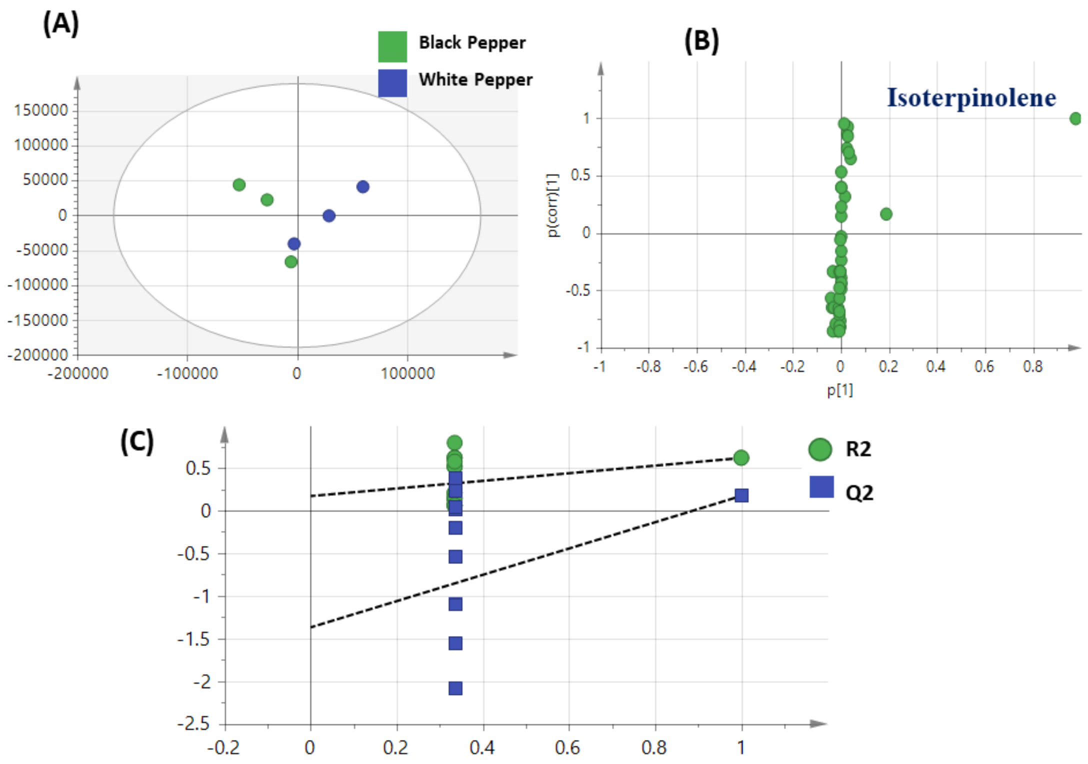

Figure S11. A) OPLS-DA score plot. B) Loading S-plots derived from modeling black pepper against white pepper as analyzed using SPME GC-MS showing the covariance p (1) against the correlation p(cor) (1) of the variables of the discriminating component of the OPLS-DA model. C) Permutation plot (n=20).

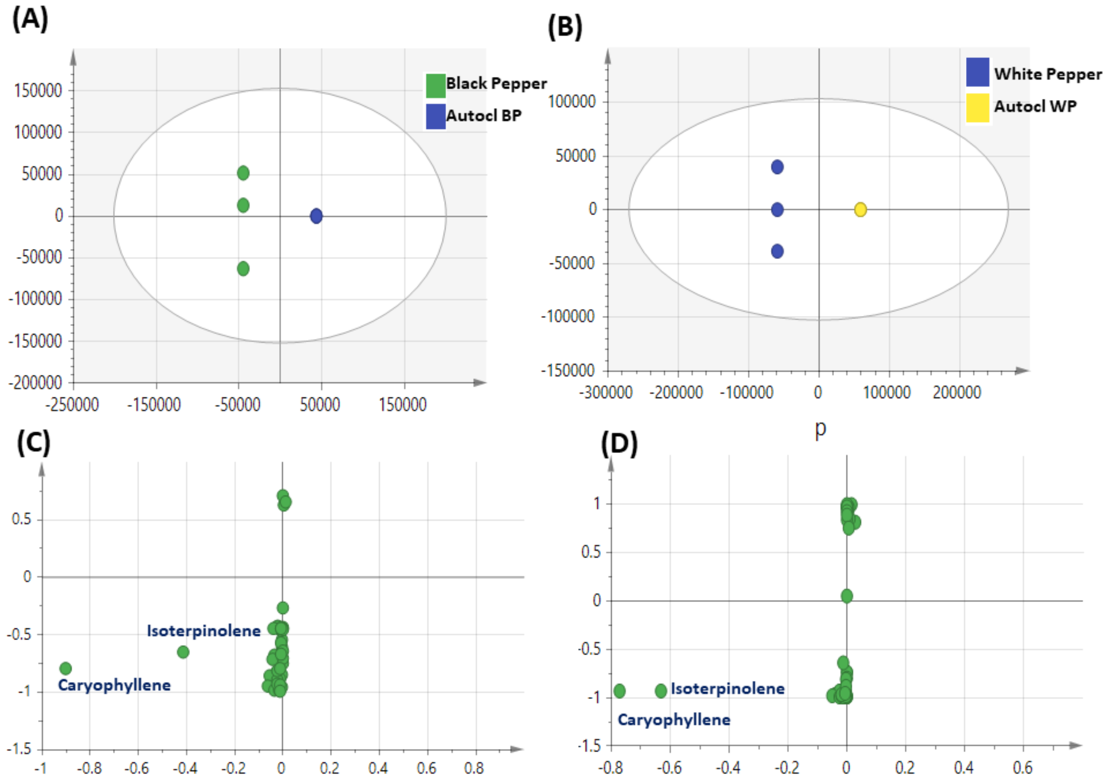

Figure S12. GC–MS-based OPLS-DA score plot derived from modeling volatile metabolites in raw and autoclaved black pepper (A), .and raw and autoclaved white pepper (**B**) alongside their respective loading plots (**C & D**) Designated variables are highlighted and identifications are discussed in the text.

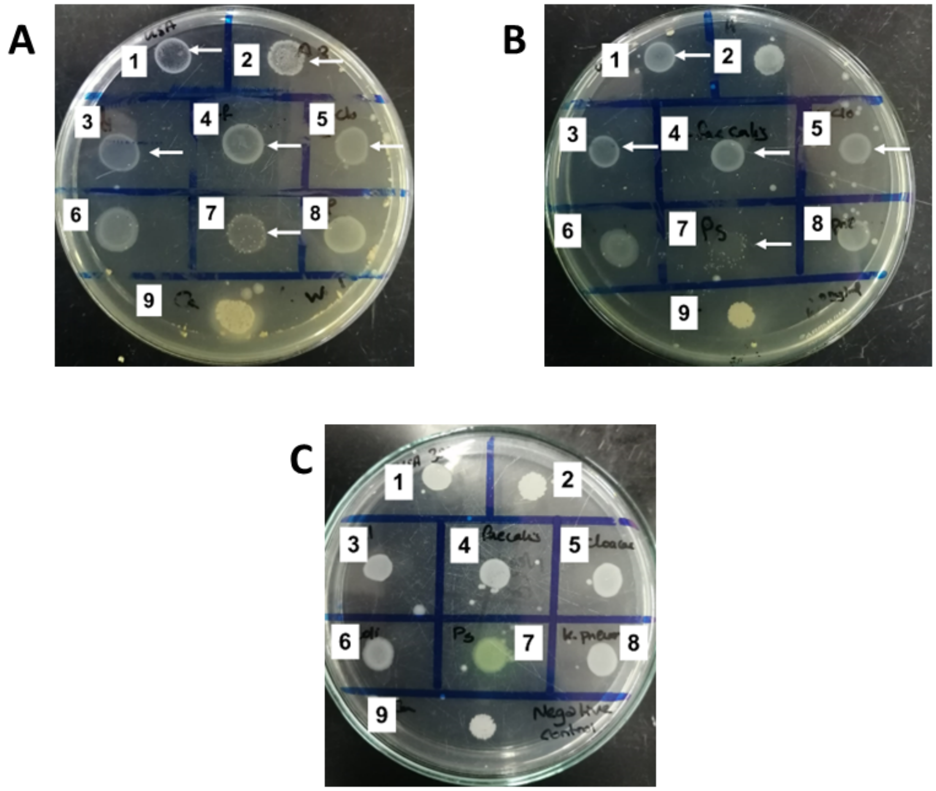

Figure S13: Inverted petri dishes showing the vapor-phase MIC experiment for (A) ground white pepper, (B) ground Black pepper, (C) growth control.

The cultures were spotted on MHA and numbered as follow: 1- MRSA *Staphylococcus aureus* USA 300, 2- *Acinetobacter baumannii* AB5075, 3- *Salmonella typhi* ATCC35664, 4- *Enterococcus faecalis* ATCC19433, 5- *Enterobacter cloacae,* 6- *Escherichia coli* ATCC87, 7- *Pseudomonas aeruginosa* PAO1, 8- *Klebsiella pneumoniae* ATCC13883, 9- *Candida albicans.* The cultured agar was upward while the ground pepper was placed downwards. The white arrows on the spots in (A) and (B) indicate a positive growth suppression. VP-MIC was determined as the least concentration of the tested sample that resulted in apparent growth inhibition of the tested microorganism when compared to the control.
